# ESCAP practice guidance for autism: a summary of evidence-based recommendations for diagnosis and treatment

**DOI:** 10.1007/s00787-020-01587-4

**Published:** 2020-07-14

**Authors:** Joaquin Fuentes, Amaia Hervás, Patricia Howlin, (ESCAP ASD Working Party)

**Affiliations:** 1 Child and Adolescent Psychiatrists, Policlínica Gipuzkoa Research Consultant, GAUTENA Autism Society, Paseo de Miramón 174, 20016 San Sebastián, Spain; 2 Child and Adolescent Psychiatrists, University Hospital MutuaTerrassa, AGAUR Clinical and Genetic Research Group IGAIN, Barcelona, Spain; 3 Emeritus Professor of Clinical Child Psychology, Institute of Psychiatry, Psychology and Neuroscience, King’s College, London, UK

**Keywords:** Autism, Diagnosis and treatment, ESCAP review

## Abstract

Across Europe, there is increased awareness of the frequency and importance of autism spectrum disorder (ASD), which is now recognised not only as a childhood disorder but as a heterogeneous, neurodevelopmental condition that persists throughout life. Services for individuals with autism and their families vary widely, but in most European countries, provision is limited. In 2018, European Society of Child and Adolescent Psychiatry (ESCAP) identified the need for a Practice Guidance document that would help to improve knowledge and practice, especially for individuals in underserviced areas. The present document, prepared by the ASD Working Party and endorsed by the ESCAP Board on October 3, 2019, summarises current information on autism and focuses on ways of detecting, diagnosing, and treating this condition.

**Electronic supplementary material:**

The online version of this article (10.1007/s00787-020-01587-4) contains supplementary material, which is available to authorized users.

## Process and methods for the development of this practice guidance

The first author (J.F.) was designated, in July 2015, as the ESCAP Autism Field Advisor, with the brief of considering the setting up of European autism guidelines. In June 2018, the Board appointed three experts (J.F., A.H. and P.H.) as the ESCAP ASD working party and instructed them to produce the Practice Guidance document. The resulting draft document was reviewed by members of the ESCAP Clinical Division and, following some revisions, was subsequently endorsed by Board of ESCAP on 03/10/2019.

The primary goal of this document is to disseminate information that can be adopted for use in regular clinical practice across Europe and to present clinicians and educators with evidence-based advice on core and minimum standards for good practice in the assessment and treatment of autistic people of all ages. It was not possible, within the scope of the present article, to complete systematic reviews and/or meta-analyses of all the issues related to autism covered here. Consequently, the aim is not to make specific recommendations for the use of particular interventions or assessment methodologies but rather to provide general guidance, based on a combination of information from randomised and non-randomised trials, expert opinion, and other existing international guidelines.

During the period from June 2018 to April 2020, a narrative review of papers published in English on evidence-based diagnostic assessments and treatments across the life cycle of people with autism^1^ was conducted. The emphasis was on European clinical research, but also included key international contributions. We focussed on clinical research updates published during the 3 years prior to submitting the final draft of this document and, in addition, we consulted existing clinical guidelines from Spain [1, 2], United Kingdom [3–5], United States of America [6, 7], Australia [8], and Scotland/United Kingdom [9].

## Diagnostic classification systems

### DSM-5

Throughout this document, the term “autism” is used as shorthand for autism spectrum disorder, as defined in the fifth edition of the American Psychiatric Association’s Diagnostic and Statistical Manual of Mental Disorders (DSM-5) [10]. DSM-5 provides standardised criteria to help diagnose autism (see Table 1) and clarifies a number of important practical and conceptual issues.

**Table 1:** Diagnostic criteria—DSM-5 [10]

*ASD diagnostic criteria*
All of the following symptoms describing persistent deficits in social communication/interaction across contexts, not accounted for by general developmental delays, must be met:
Problems reciprocating social or emotional interaction, including difficulty establishing or maintaining back-and-forth conversations and interactions, inability to initiate an interaction, and problems with shared attention or sharing of emotions and interests with others
Severe problems maintaining relationships—ranges from lack of interest in other people to difficulties in pretend play and engaging in age-appropriate social activities, and problems adjusting to different social expectations
Non-verbal communication problems such as abnormal eye contact, posture, facial expressions, tone of voice and gestures, as well as an inability to understand these
Two of the four symptoms related to restricted and repetitive behaviour need to be present:
Stereotyped or repetitive speech, motor movements, or use of objects
Excessive adherence to routines, ritualized patterns of verbal or nonverbal behaviour, or excessive resistance to change
Highly restricted interests that are abnormal in intensity or focus
Hyper or hypo-reactivity to sensory input or unusual interest in sensory aspects of the environment

Firstly, although in certain European countries, autism still remains in the category of “psychotic disorders” [11], DSM-5 makes it clear that autism is not a psychotic disorder. Instead, autism is included within the domain of “Neurodevelopmental Disorders”. This is a group of conditions, usually evident in the pre-school years, characterised by specific developmental impairments in cognitive, psychological, communication, social, adaptive, and/or motor functioning. The classification also includes intellectual disability (intellectual developmental disorder), communication disorders, attention-deficit/hyperactivity disorder, specific learning disorder, and motor disorders.

Secondly, DSM-5 no longer divides autism into separate disorders [i.e., autistic disorder, Asperger’s disorder, childhood disintegrative disorder, and pervasive developmental disorder not otherwise specified (PDD-NOS)] as was previously the case in DSM-IV-TR [13] and International Classification of Disorders-10 (ICD-10) [14]. This reflects the current scientific consensus that these can be incorporated within a single, dimensional condition, with variable levels of symptom severity in two core domains: (i) deficits in social communication and social interaction; (ii) restricted repetitive behaviours, interests, and activities. If this second domain is absent, the condition is classified as Social Communication disorder.

Concerns have been raised [15, 16] that some individuals, who had previously been diagnosed with Asperger’s Disorder, would lose their autism diagnosis under the revised DSM-5 criteria and could be denied support, especially in countries where a specific medical diagnosis is required for intervention. However, DSM-5 explicitly states that “Individuals with a well-established DSM-IV diagnosis of autistic disorder, Asperger’s disorder, or pervasive developmental disorder not otherwise specified, should be given the diagnosis of autism spectrum disorder”. Nevertheless, there are individuals who may never fully meet current criteria but who are still negatively affected by their symptoms. There are also individuals who, while meeting core criteria as children, later improve to such an extent that they no longer meet full diagnostic criteria, although their autism does not completely disappear. DSM-5 makes no provision for such individuals who, because they do not meet all diagnostic criteria, may risk losing access to any specialist support or provision. ICD-11 criteria (see below), however, do account for those individuals.

Thirdly, DSM-5 allows for further distinctions according to severity level. Thus, clinicians and researchers are encouraged to use “Specifiers” to identify individual characteristics such as: with or without accompanying intellectual impairment; with or without language impairment; associated with known medical or genetic condition or environmental factor, or with another neurodevelopmental, mental, or behavioural disorder. Severity is also rated according to the amount of support needed. This ranges from level 1 (needing some support) to 3 (needing very substantial support). However, the manual is unclear on what exactly is meant by “some”, “substantial” or “very substantial” support.

DSM-5 also notes that symptoms must be present in early childhood, but may not become fully manifest until social demands exceed capacities. Symptoms need to be functionally impairing and not better described by another DSM-5 diagnosis.

### ICD-11

The World Health Organization (WHO) released the latest International Classification of Diseases (ICD-11) in June 2018 [17]. ICD-11 will come into effect on the 1st of January 2022. There are close parallels between DSM-5 and ICD-11 classifications of autism. This is important, since, while clinicians and researchers in many countries use the DSM, the ICD is the coding system officially used in most countries of the world. Unlike the WHO system, DSM does not depend on complex international agreements and procedures and, thus, has been updated and revised more frequently than ICD. In the past, this has resulted in (sometimes substantial) differences between the two systems, leading to clinical and research complications; fortunately, this is no longer the case.

Like DSM-5, ICD-11 includes autism in the category of Neurodevelopmental Disorders. This comprises disorders that arise during the developmental period and involve significant difficulties in the acquisition and execution of specific intellectual, motor, or social functions. Although behavioural and cognitive deficits are present in many other disorders that also arise during the developmental period (e.g., Schizophrenia and Bipolar disorder), only disorders whose core features are neurodevelopmental (i.e., identified by early and continuing cognitive, social, and behavioural deficits) are included in this grouping. The ICD-11 classification of Developmental Disorders includes essentially the same conditions as DSM-5: i.e., Disorders of intellectual development, Developmental speech and language disorders, ASD, Developmental learning disorders, ADHD, Stereotyped movement disorders, Tics, and other specified or unspecified neurodevelopmental disorders.

As in DSM-5, ICD-11 groups autism symptoms into two core domains: (i) persistent deficits in the ability to initiate and sustain reciprocal social interaction and social communication, and (ii) a range of restricted, repetitive, and inflexible patterns of behaviour and interests. Deficits must be sufficiently severe to cause impairment in personal, family, social, educational, occupational, or other important areas of functioning. These difficulties are usually observable across all settings and are a pervasive feature of the individual’s functioning, although symptom severity may vary according to social, educational, or other contexts. ICD-11 also notes that individuals with autism may vary across the whole spectrum of intellectual and language functioning, but instead of the descriptive severity classification of DSM-5, the following subdivision is proposed (see Table 2). The validity and stability of these combinations are still to be demonstrated.

**Table 2:** Subdivision of disorders in ICD-11 [17]

ASD (6A02)		
	Disorder of intellectual development	Impairment of functional language
6A02.0	*Disorder not present*	*Not present or Mild impairment*
6A02.1	Disorder present	*Not present or Mild impairment*
6A02.2	*Disorder not present*	Impairment present
6A02.3	Disorder present	Impairment present
6A02.4	*Disorder not present*	Absence of functional language
6A02.5	Disorder present	Absence of functional language

There are two other additional categories (6A02.Y/6A02.Z) for “other” and “unspecified”

– no criteria given

Unlike DSM-5, ICD-11 does not include hyper- or hypo-reactivity to sensory input, or unusual interests in sensory aspects of the environment. However, the section on developmental language disorder now includes a category characterised mainly by impairment in pragmatic language, which mirrors the DSM-5 social communication disorder.

The development of the DSM and ICD classification systems has been crucial in clarifying diagnostic criteria and standardising diagnostic practice. Nevertheless, it is important to remember that such systems describe and classify disorders, not people. WHO in their autism spectrum disorder summary states: “It is important that, once identified, children with an ASD and their families are offered relevant information, services, referrals, and practice according to their individual needs” [18].

## Prevalence

Across the world, there has been a dramatic rise in the numbers of individuals diagnosed with autism in recent decades, with prevalence estimates increasing from around 0.04% in the 1970s to 1–2% currently. However, epidemiological figures vary widely (e.g., see the interactive global prevalence map at https://prevalence.spectrumnews.org) and comparisons between studies, or over time, are compromised by very different methodologies, variable sampling procedures, and many other challenges, in particular, inconsistencies in case definition and diagnostic criteria. Moreover, figures based on direct examination produce very different, and generally much lower, estimates than those obtained from case records or derived from instrument cut-off scores [19].

Taking account of all these factors, plus a lack of data from much of the world, especially from low- and middle-income countries, WHO estimated that the global prevalence of autism in 2012 was about 0.6% [20]. More recent estimates are higher, most likely due to increased public and professional awareness, inclusion of cases without intellectual disability, and improved diagnostic practice and referral opportunities. Thus, USA surveillance data (2016) suggest a prevalence of 1.85%, although there were significant differences among the 11 sites included in this survey [21]. Other studies report estimates exceeding 3% [22].

Even taking the most conservative estimate, it is now generally agreed that the prevalence of autism is at least 1%. To put this figure in perspective, this means four and a half million people in the European Union have autism. And, for each one of these individuals, at least another three per household—parents and one sibling—will have their lives directly affected. This means 18 million people in the EU alone—a significant part of our population.

## Early detection

Unfortunately, in most countries, advances in awareness of autism have not been accompanied by improvements in the age of diagnosis. Parents typically have concerns about their child’s development by the age of 18–24 months, but a review of 42 studies found that the mean age for diagnosis in developed countries varied from 38 to 120 months [23]. In less-developed countries, or in populations without medical insurance or access to free healthcare, age of diagnosis is likely to be much later.

Although speech delay is not specific to autism [24], delays in babbling /talking are often the first signs to give rise to parental concerns. Other early symptoms include delays in pointing/gesturing, responding to own name, and poor eye contact [25]. Difficulties in social interaction with peers and/or unusual repetitive behaviours may not be apparent in the first 2–3 years, and thus, their absence in very young children should not rule out a possible diagnosis. The US Centers for Disease Control and Prevention Web Page [26] list some of the following characteristics as possible “Red Flags” for autism in toddlers (see Table 3).

**Table 3:** "Red Flags" for autism in children [26]

Does not respond to their name by 12 months of age
Does not point at objects to show interest (point at an airplane flying over) by 14 months
Does not play “pretend” games (pretend to “feed” a doll) by 18 months
Avoids eye contact and want to be alone
Has trouble understanding other people’s feelings or talking about his or her own feelings
Has delayed speech and language skills
Repeats words or phrases over and over (echolalia)
Gives unrelated answers to questions
Gets upset by minor changes
Has obsessive interests
Flaps their hands, rocks their body, or spins in circles
Has unusual reactions to the way things sound, smell, taste, look, or feel

### Early decline

Over 20 years ago, a number of case studies described sudden autistic regression (loss of language, social, cognitive, and motor skills) in children between 1 and 2 years of age, who had previously shown a period of normal development. It was wrongly suggested that these cases were associated with external psychosocial or medical events, including vaccinations [27]. Subsequent research indicated that severe and sudden regression in children who had previously shown typical development across multiple domains is rare [28] and, if it occurs, may be associated with Landau–Kleffner syndrome, an acquired epileptiform aphasia identified by clinical and EEG data [29]. Retrospective clinical data also indicated that many children who were reported to have regressed had existing prior socio-communicative difficulties [30]. However, recent prospective studies indicate that the onset of autism is often preceded by a subtle decline, during the first 24 months, in trajectories of key social and communication behaviours, suggesting that a regressive onset pattern may occur more frequently than previously recognised [31].

### Early identification of autism

There is no single measure that can be recommended for the early detection of autism [3], but several commonly used screening instruments are widely available (see Supplementary Material Tables S1, S2). While these vary in terms of age range, length, and source of data (i.e., direct observation or responses from parents or professionals), many are brief, taking 5–10 min to complete, and most are inexpensive or free. Some are available in different languages and, while the majority focusses on children, a few are also appropriate for adults. Although sensitivity is generally higher than specificity (that is they identify other developmental problems as well as autism), from a public health viewpoint this can be considered a positive asset, since they identify cases requiring the early developmental support.

It is important to be aware that the purpose of screening instruments is to alert physicians to the possibility of autism; they are not designed to confirm diagnosis. Moreover, not all children with autism do score positively on these instruments and failure to meet cut-off criteria does not necessarily mean that an autism diagnosis should be excluded. If other sources of information indicate developmental delay or disturbance a full diagnostic assessment is still warranted.

Currently, despite ongoing research efforts (https://www.aims-2-trials.eu/our-research/biomarkers), there are no specific biomarkers that can reliably identify children at risk of autism, and thus, it is important to make the most efficient use of existing identification procedures. Although the American Academy of Pediatrics [32] has recommended autism-specific screening for all children at 18 and 24 months, as well as regular developmental surveillance at each well-child visit at 9, 18, and 30 months, the United States Preventive Services Task Force [33] suggests that there is insufficient evidence to recommend screening for children in whom “no concerns of ASD have been raised by parents, other carers or other health professionals”. They conclude that, at the present time, more research into the potential benefits of screening is needed; meanwhile, clinicians should listen carefully to parents’ concerns and use validated tools to assess the need for further diagnostic or other services.

Earlier recognition of autism symptoms could also be improved by greater awareness of risk factors among professionals. Individuals at heightened risk of autism include: siblings of already identified cases; babies born to older fathers and older or very young mothers; history of sub-optimal pre-or perinatal development (e.g., medication use in pregnancy, maternal obesity, hypertension, or infection) [34]. The risks of autism are also increased among children with a range of genetic conditions [35] and/or those with other disorders, such as anxiety and mood disorders [36], attention-deficit/hyperactivity disorder [37], and obsessive–compulsive disorder [38]. However, given the reality of services in many European countries, and increased awareness of autism among parents, the simplest and most effective mechanism for improving detection could be to ensure that care professionals respond much more rapidly to parental concerns, especially about early delays/abnormalities related to socially interactive behaviours such as gaze, smiles, and social vocalizations [39]. Teachers can also be a crucial source of information, especially in those areas of Europe where early schooling (i.e. from age 2 + years) is the rule [40]. They are in a unique position to identify the relative strengths, difficulties and differences of these children, compared with “typically developing” pupils, and to refer them for appropriate assessment when necessary.

Regrettably, despite improved knowledge about the early signs of autism, many families who seek diagnosis are still met by a “wait and see” or “he/she will grow out of it” response, by family doctors and paediatricians. And, even when children at potential risk of autism are recognised, long waiting lists are common, even in the best served communities. This calls for improving available diagnostic and assessment services across the whole of Europe. Early identification of children at risk of autism is important to offer support to families and facilitate access to appropriate education and treatment. While the long-term benefits of early intervention programmes (see “Treatment”) remain uncertain, there is good evidence that intervention in the early years can help to enhance social communication skills, minimise symptom severity, optimise child development, and improve parental stress and well-being. Thus, early identification of children at risk of autism is important to offer support to families and facilitate access to appropriate education and treatment [41–43].

## Diagnosis

The essential aim of a diagnostic assessment for autism is to ensure that individuals, and their families, are provided with appropriate support and guidance. NICE UK guidelines [3] state: “the focus of assessment should not only be on diagnosis but should also consider the risks a person faces, as well as their physical, psychological and social functioning…. in all cases the central aim is to identify need for treatment and care”. For this reason, the diagnostic process must include not only assessment of autism symptomatology, but should systematically explore developmental history, current cognitive and language functioning, social and family background, and possible underlying genetic mechanisms. Clinical examination is also needed to identify any accompanying neurodevelopmental, physical, and/or sensory difficulties. Interested readers can review a recent and comprehensive German Guideline on Diagnosis [44].

Autism is a highly complex and heterogeneous condition that evolves with age. Hence, the diagnostic team should be multi-professional, involving, at the very least, medical (psychiatric and/or paediatric) and psychological (clinical or educational) input from clinicians with training and experience in developmental disorders. Depending on local availability, expertise from other professions (speech and language therapy, child or adult neurology, education, occupational therapy, physiotherapy, etc.) will also enhance the diagnostic process.

Diagnosis requires not only direct individual assessment but also information from carers (usually the parents) and all others who play an essential role in the person’s life (e.g., teachers of school children; partners or siblings of older individuals). The amount of detail collected during the diagnostic process will vary according to local provision, but at a very minimum should include the following components: (see Table 4) [3]

**Table 4:** Minimum assessment for autism in children [3]

Clinical history
Identification of autism symptomatology
Assessment of developmental level and expressive and receptive language skills
Physical and sensory examination (hearing and vision; neurological screening, etc.)
Assessment of emotional or behavioural difficulties
Information on socio-environmental factors

The diagnostic assessment should begin with a detailed clinical history to establish the individual’s developmental trajectory, medical history, developmental level, and relevant family or social factors (see Table 5) [5]*.*

**Table 5:** Key elements for autism of a clinical history [1]

Family history	Nuclear and extended family history of similar problems and/or neurodevelopmental disorders. Age of parents. Presence of siblings
Pre/perinatal antecedents	Pregnancy and delivery. Previous use of medications by mother. Birth weight, APGAR Scores, screening of metabolic or congenital disorders
Developmental history	Motor, communication, and social milestones. Sleeping, eating, sphincter control. Main concerns or early unusual behaviour reported by carers
Medical history	Medical and genetic conditions; auditory, visual, sensory difficulties; neurological problems
Family and psychosocial	Functioning of the child in family, school and social environments. Challenges experienced and support provided. Family situation and status
Diagnosis and interventions	Outcome of previous consultations, review of reports, assessments or interventions available from social, health and/or educational resources

*Autism symptomatology* Identification of autism symptoms should include direct observation of the individual as well as information from carers. A number of standardised assessment instruments have been developed to assess the presence and/or severity of autism (see Supplementary Table S3), although most are costly and/or require specialist training. While the use of these supporting instruments is highly recommended, it is important to recognise that none should be used, in isolation, to make the diagnosis. This requires expert clinical judgement based on information gathered from all relevant sources.

*Developmental assessments* Autism is frequently accompanied by cognitive and language difficulties that have a major impact on functioning and prognosis. Moreover, many children with profound intellectual difficulties show patterns of development that are similar to autism; hence, developmental information is crucial for differential diagnosis as well as for planning appropriate educational provision and intervention.

A wide range of standardised tests is available for the assessment of intellectual, language, and functional ability (see Supplementary Table S4). Although some are expensive and require specific training, informant-based measures can be used to assess individual profiles of expressive and receptive language, social competence, and functional abilities across a wide age range. Since developmental profiles in autism are often very uneven, an overall IQ figure may not adequately reflect an individual’s functional ability. Instead, the focus of assessment should be to identify specific areas of relative strengths and weakness and to recommend appropriate strategies to overcome, minimise, or circumvent areas of difficulty.

### Common co-occurring conditions

*Physical and sensory problems* Autism is associated with a range of medical conditions, including hearing and visual impairments, epilepsy, and other neurological disorders. Consequently, the diagnostic process should include thorough physical and sensory assessments together with a standard neurological examination, if possible conducted by a neuropaediatrician in the case of children. If no specific deficits are identified, there is usually no clinical need for additional EEG and/or neuroimaging testing [1, 3, 45]. However, if there are causes for concern (such as epilepsy or unexplained loss of skills), more detailed neurological testing is required.

*Genetic disorders* In families with one autistic child, there is a 10% recurrence risk of autism and a 20–25% risk of other neurodevelopmental disorders in siblings. The recurrence risk increases to 36% in families with two autistic children [32]. Autism is associated with a number of genetic disorders, but relatively few have a monogenic cause; for example, fragile X syndrome and tuberous sclerosis complex together are estimated to account for < 10% of cases of autism (See Lord et al. [46] for a very helpful summary of genetic mechanisms). More commonly, de novo, rare, heterozygous mutations of single base (nucleotide) or/and sub microscopic segments of DNA (copy-number variations or CNVs) are found and more than 100 genes have been identified in autism. Advances in genetic technology have now made it possible to identify genetic aetiologies in 25–35% of people with autism [8].

Identification of possible genetic causes is important to ensure access to genetic counselling; to identify possible risks in other family members, and for the provision of appropriate medical care (e.g., for identifying the risk of associated disorders such as heart or cerebral dysfunction) [32]. Basic assessment should include intergenerational family history, physical examination, screening for pathogenic mutations in the MECP2 gene (47), Fragile X testing in males, and in females if indicated by family history and/or phenotype; and PTEN gene analysis [48] if other symptoms suggest that these are warranted (see Supplementary S5 for protocol [49] for genetic investigation). Whole-exome or whole-genome sequencing for autism is becoming increasingly used in parts of North America and Europe and could soon become the standard of care in some countries [50]. The rapidly declining cost of these techniques [51] and international recruitment of large samples [52] have resulted in the identification, by exome sequencing, of 102 risk genes for autism: 49 are associated with co-occurring neurodevelopmental delay, 53 with autism alone.

*Emotional and behavioural problems* Individuals with autism have a significantly increased risk for many other disorders including attention-deficit/ hyperactivity disorder, irritability, aggression, sleep problems, and mental health conditions, particularly anxiety and depression [53]. An elevated rate of autism symptoms has also been found in children with Tourette syndrome [54]. Other co-occurring disorders such as post-traumatic stress disorder, eating disorders, gender dysphoria, and substance abuse have received relatively less empirical study [55, 56], but all these problems can have a significant and negative impact on functioning and quality of life, and greatly increase parental/carer stress. As in mental health services generally, standard diagnosis involves history-taking; mental state examination, taking into account developmental level; gathering information from multiple sources, and assessing the family, educational, and social context. Ideally, diagnostic formulation is based on clinical interview and, if appropriate, the use of relevant questionnaires/rating scales [57]. A formal categorical diagnosis should then lead to treatment recommendations.

Nevertheless, in the case of autism, the identification of an emotional or behavioural disorder is just the beginning of a personalised assessment process and intervention plan [58]. Determining the reasons for and nature of such problems requires careful observation across different settings; information from multiple sources, and systematic exploration of factors that may cause or exacerbate symptoms. These can include inappropriate environmental demands, lack of structure, sensory overload, painful medical conditions, difficulties in recognising or dealing with emotions, lack of effective communication, difficulties coping with transitions or sudden change, or the pressures of social situations. Many behaviours, including behaviours that challenge, occur, because they serve a function and/or produce an outcome for the individual [59]. A “functional analysis” of underlying factors makes it possible to formulate hypotheses about potential causes of, and solutions to problems. A temporary action plan can then be implemented; the effects of this should be carefully reviewed and treatment subsequently modified as necessary.

### Socio/environmental considerations

Although autism is not caused directly by adverse family or environmental circumstances, family discord, and parental illness (mental or physical), social and economic deprivation can limit the chances of receiving adequate support, exacerbate existing problems, and worsen prognosis. Thus, the diagnostic assessment should also include information on any circumstances that may have a negative impact on the autistic individual and/or his or her family.

### Assessments in adulthood

The diagnosis of autism in adulthood presents additional challenges. Almost all diagnostic instruments were initially developed for children, but in adulthood, it is sometimes difficult to obtain early developmental data. Hence, clinical judgment frequently has to rely on self-report and/or data from other sources (e.g., siblings, friends, and partners) to ensure that all relevant information is included (see [60] for review). Moreover, in adulthood, autism symptoms may be less evident than in childhood, especially in cognitively able individuals who have developed ways of circumventing or disguising some of their difficulties. Additional problems, such as depression or anxiety further complicate the clinical picture. NICE [5] lists a number of signs that are suggestive of autism in adulthood (see Table 6). Information on past contact with child services, indications of earlier neurodevelopmental problems, and assessment of current functioning (especially when functional ability is out of synchrony with cognitive level) can also help to clarify diagnosis.

**Table 6:** Recognising autism symptoms in adults [5]

Adults of higher IQ	
One or more of the following:	Persistent difficulties in social interaction Persistent difficulties in social communication Stereotypic (rigid and repetitive) behaviours, resistance to change or restricted interests and
One or more of the following:	Problems in obtaining or sustaining employment or education Difficulties in initiating or sustaining social relationships Previous or current contact with mental health or learning disability services A history of a neurodevelopmental condition (including learning disabilities and attention-deficit hyperactivity disorder) or mental disorder
Adults with moderate or severe learning disability (using information from a family member or carer)	
Two or more of the following:	Difficulties in reciprocal social interaction including: Limited interaction with others (e.g., being aloof, indifferent, or unusual) Interaction to fulfil needs only Naïve or one-sided interaction Lack of responsiveness to others Little or no change in behaviour in response to different social situations Limited social demonstration of empathy Rigid routines and resistance to change Marked repetitive activities (for example, rocking or finger flapping) especially when under stress or when expressing emotions

### Diagnosing autism in women and girls

The male-to-female ratio in autism has generally been estimated as around 3–4:1 [61], with some studies, suggesting that the male:female ratio could be as high as 8–9:1 in individuals within the average/above average range of intellectual ability [62]. However, there is now growing awareness that many females with autism remain undiagnosed for a number of reasons [63]. Thus, the manifestation of symptoms in girls and women with autism may be atypical. In particular, social and communication deficits are often more subtle than in males and females may be more adept at “masking” or “camouflaging” their differences [64–66]. Compared with boys, girls are more likely to try to socialise, and frequently, they have one or two friends; they also tend to develop more imaginative play, use more emotional language, and show less repetitive behaviour. Special interests, too, often have a more social content. Current autism diagnostic assessments make no allowance for possible gender differences and identifying autism in females may require much more focused and detailed clinical enquiry. Absence of, or very delayed diagnosis, lack of help for their difficulties, and the stress of trying to appear normal, increase the risk of girls developing emotional and other problems (including eating disorders) [67], while their naivety in social relationships exposes them to victimisation, teasing and sexual aggression or abuse.

The diagnosis of autism in adult women who have not received a diagnosis in childhood is particularly complicated. Many are misdiagnosed with a variety of mental health conditions, such as borderline personality disorders. Often, the clinician lacks access to informants who have known the individuals since childhood and the high emotionality, difficulties of expression, and introspection of some women with autism means that they can have problems in explaining their difficulties or feelings. It is important that professionals do not immediately reject a diagnosis of autism in females, even if symptoms are below the diagnostic threshold. Instead, it may be necessary to see the individual over several sessions before reaching a definitive diagnosis [68].

### Limitations of standard diagnostic procedures

The assessment procedures described above will, in the majority of cases, provide valid information on which to determine diagnosis of autism and any co-occurring conditions. However, clinicians should also be aware that there are circumstances when standardised procedures may be inadequate or misleading. For example, current autism diagnostic instruments were developed mainly with co-operative, middle-class families. Their utility with economically deprived, socially isolated, minority ethnic, or racial groups remains uncertain. When exploring mental health problems, it is important to recognise that standardised psychiatric clinical instruments have not, in general, been validated for this population. Moreover, many autistic people, of all ages, find it very challenging to self-reflect or to describe feelings or emotions, especially within the framework of a face-to face clinical interview, while proxy reports by parents or carers may fail adequately to reflect individuals’ real emotional state.

It is also well established that the risk of autism is significantly increased in individuals with severe sensory problems (profound deafness and congenital blindness); in those with profound intellectual impairment, and in many genetic conditions (e.g., Fragile X, Down syndrome, Rett syndrome, Cornelia de Lange syndrome, Tuberous Sclerosis Complex, Angelman Syndrome, Neurofibromatosis Type 1, Noonan Syndrome, Williams Syndrome, and 22q11.2 Deletion Syndrome) [35]. Making the additional diagnosis of autism in conditions where social communication difficulties and stereotyped/repetitive behaviours are part of the behavioural phenotype clearly complicates the diagnostic process. Families of children with an identified genetic or sensory disorder also often report difficulties in obtaining a diagnosis of autism, because this tends to be overshadowed by the primary medical diagnosis [69].

### Sharing diagnostic information

Receiving a diagnosis of autism can be one of the most important events in the lives of an individual and his or her family, and the manner in which diagnostic information is conveyed may have profound and long-term effects. All families deserve a full explanation of how the diagnosis has been reached and what the implications may be in terms of future provision and support. If, for any reason, it is not possible to arrive at a precise diagnosis (or the conclusion is that the individual does NOT have autism), this, too, must be fully explained. Information must be provided clearly and precisely, using language that is appropriate for the family’s social and educational background. It is particularly important for clinicians to bear in mind that, not uncommonly, one or more family members will also share characteristics of autism, so communication at all times must take this into account. It is essential to convey the diagnosis with empathy and to acknowledge the likely emotional impact of this on the family [1].

Many families find it very difficult fully to understand or process the information which they are given at the time of diagnosis. Thus, they need adequate opportunity to ask questions, preferably at a follow-up appointment when they have had time to reflect on what they have been told. Families should also be informed that, post-diagnosis, they will receive a comprehensive, written report, detailing the conclusions of the assessment, relevant sources of information, and arrangements for further support. The written report will also enable them to share the key findings and recommendations with the services that will subsequently be responsible for providing this support, as well as with relevant family members and friends [1].

## Prognosis in autism

For many families, the first thing they want to know, after receiving the diagnosis, is “What will his/her future be?” “Will my child go to regular school; get a job; get married; have children; live independently?”. Unfortunately, a diagnosis of autism, of itself, provides little information about overall current or future functioning. This may be improved somewhat by the inclusion, as in ICD-11 [17], of details about cognitive and language ability, but, nevertheless, a diagnosis made in very early childhood cannot reliably predict functioning in later childhood, adolescence, and adulthood. For example, some individuals with autism and good intellectual ability achieve very highly as adults; others remain heavily dependent on family and social services for support throughout their lives; some individuals with mild intellectual disability go on to live very productive lives; others who showed severe behavioural disturbance or social avoidance as children become well-functioning and sociable adults

Despite the variability, research evidence suggests that most autistic individuals with moderate–severe cognitive impairments, especially if they remain without useful language, will continue to need specialist educational provision as children, and support with employment and daily living in adulthood. For individuals of higher intellectual ability (i.e., IQ > 70), the future frequently depends on how much support is provided to enable them to access school, college, jobs, and independent living and be fully included in society. Follow-up studies, from childhood to adulthood, generally indicate that the severity of core autism symptoms decreases over time and many individuals show marked improvements in social and communication skills as they grow older. Among the strongest predictors of a positive outcome are the development of language and having a non-verbal IQ in or around the average range [70, 71]. Within this more cognitively able group, there are a small number of adolescents or young adults who no longer meet diagnostic criteria and show no clear remaining symptoms of autism. However, research suggests that they continue to show subtle difficulties in social understanding, pragmatic communication, attention, self-control, emotional maturity, and psychiatric morbidity [72].

Overall, it is evident that outcome in adulthood, even for individuals of average IQ or above, is generally poor [73]. Access to higher education and employment is limited and fewer than a third of adults are in full-time employment. The majority remains dependent on families and/or state benefits for support and only a minority develops close, long-term relationships. There are a few evidence-based interventions for adults, and specialist help and support are particularly limited for individuals of average or above intelligence.

Very many autistic adults experience mental health problems that can greatly impair functioning and reduce their quality of life. It is suggested that the constant demands of ‘fitting-in” to a non-autism world; the stress of “camouflaging” to avoid being perceived as different [60] and the absence of appropriately structured support or daily activities all contribute to high levels of stress, anxiety, and depression. Physical problems and chronic health disorders are also more common than in the general population [74, 75] and recent studies highlight the risk of premature mortality [76–78]. Premature mortality is particularly high in autistic adults with intellectual disability and epilepsy. In individuals of average or above average IQ, suicide is a significant cause of premature death [76].

### Autism in older adults

Relatively little is known about the lives of older individuals with autism (i.e., 60 + years) and there is little information on whether, compared with the general population, these individuals show greater cognitive deterioration with age, or whether they show areas of relative preservation or strength. However, there is some evidence that, while verbal memory in autism shows a similar decline as in “typical” ageing, visual and working memory skills tend to be better preserved [79]. There may also be less deterioration in mental health and in overall quality of life in older adults with autism than in the general elderly population. There is some suggestion, too, that the risks of developing Alzheimer dementia may be reduced [80].

## Treatment

As highlighted throughout this document, autism is a highly heterogeneous condition. Although, for many individuals, autism results in significant impairments and restrictions on their lives, others view their autism as part of the normal diversity of human nature. They do not wish to be considered as having a disability, but instead stress the positive aspects of individual differences for the successful adaptation of the species [81]. Research studies also indicate that the characteristics of autism lie within a continuous distribution of social communication difficulties and repetitive or restrictive patterns of behaviour as found in the general population [82, 83]. Moreover, ICD-10 and ICD-11 criteria stipulate that if symptoms cause no clinically significant impairment in emotional, social, occupational, or other important areas of functioning, no diagnosis is merited. Nevertheless, very many individuals, and their families, will need the support of paediatric, educational, or child and adolescent mental health services, or from adult psychiatric clinics. The nature, intensity, and extent of such help will differ for each individual, and will vary with age and ability, as well as the physical, emotional, and social environment in which they live.

Many claims for treatments for autism have little or no scientific basis, but, in recent years, there has been a significant increase in the numbers of randomised-controlled trials [7], especially for very young children. There is now strong evidence for the effectiveness, and in some cases of the longer term impact, of interventions that focus on early parent–child interactions [84–86]. There is also evidence that interventions conducted in highly resourced settings can be modified to meet the needs of individuals in other cultures and more economically deprived settings [87]. Nevertheless, the scope of RCTs remains limited in terms of the range of interventions tested and the ages and characteristics of participants [88]; the number of trials in low resource countries also remains very small [89]. Moreover, no intervention works for everyone. A recent review [46] highlights that the quality of clinical trials in autism lags far behind much other research and, given the wide heterogeneity of autism, even in the context of significant treatment effects, “reliable predictors of (individual) response to treatment have not been demonstrated”. The authors of that review note that in the majority of countries, including those of high-income, most intervention is conducted by non-specialists, and thus, parents and practitioners have “no options other than what is available and sometimes marketed their region”. This leaves clinicians, especially those working with older children, adolescents, or adults with very little practical guidance on how best to assess, advise, or intervene with their patients or clients.

For the purpose of the present guidance, therefore, the ESCAP Working Party, endorsed by ESCAP, has taken the approach of combining information from randomised and non-randomised trials, expert opinion, and other existing international guidelines.^2^ Our goal is to offer professionals in Europe practical guidance for supporting individuals with autism and their families. On this basis, the following, ten general principles are recommended.

### General principles

#### *Pre-treatment assessment*

For any family with a child with special needs, early support is crucial to improve parental confidence and competence, and may also minimise the escalation of later problems. Thus, even if families need to wait for a formal diagnosis of autism, detailed assessments of the child’s skills and difficulties across multiple domains can inform general approaches to management based on the child’s individual profiles of skills and difficulties across multiple domains. Objective assessments, even of a simple kind, are essential to place observed behaviours within a developmental framework, thus serving as a baseline for planning interventions and monitoring subsequent progress.

#### *Each individual, each family is unique*

It is important to base diagnosis and assessment on reliable and valid measures, and to use evidence-based practice as far as possible. Nevertheless, conclusions from standardised assessments, and recommendations for treatment and support, must always take into account the individual’s profile of strengths, difficulties, and needs, and the family and social context in which he or she lives. It is also important to be aware that individuals, families, and circumstances change with age and thus intervention and support must be adapted to take account of these changes.

#### *Focus on individual strengths, not just limitations*

Autism is typically characterised by a very uneven profile of strengths and difficulties. Identifying and fostering individual strengths can help to provide a much more positive environment for autistic people and their families. Areas of strength can also be used to circumvent or substitute for areas of relative weakness. For example, visual communication strategies can be effective for individuals with good visual skills but limited verbal ability. Special interests (e.g., in numbers, facts, computing, etc.) can be used to improve academic or practical skills and to foster social contacts.

#### Intervention should be based on a “functional analysis” of behaviour

This involves taking account of all the factors that may be limiting an individual’s ability or quality of life and developing testable hypotheses about the potential functions of observed behaviours. This makes it possible to (i) identify potential underlying causes of difficulties; (ii) help individuals to acquire alternative and more effective/acceptable means of influencing their environment; (iii) help to develop the skills needed to improve quality of life.

#### *Focus on making the environment more “autism friendly*”

Functional analysis should also help to identify environmental factors (social, sensory, cognitive, physical, etc.) that may be limiting progress and/or quality of life. Assisting others to view the environment “through the eyes of” the autistic person, and designing ways to reduce environmental stress, is crucial. Even very minor environmental changes can have major effects on behaviour and individual well-being.

#### *Effective treatment is not determined by a fixed number of hours or sessions of intervention*

Individuals with core difficulties in social relating, communicating, and imagination face significant challenges. Treatment should not be based on a prescribed number of hours or sessions per day or week of therapy. Instead, the aim of therapy should be to ensure that all possible opportunities during the day are used to facilitate progress and minimise difficulties.

#### *Reassess the professionals’ role*

The clinician or therapist is not the main source of intervention. Their role is to serve as a coach for the key people directly involved in the individual’s life—i.e., parents, teachers, other family members, support workers, employers, etc. Whenever possible, and as recommended by NICE [3, 5] and other guidelines, each individual should have a case-coordinating professional (often called “keyworker” or “case manager”), who can be of any child and adolescent mental health discipline, and whose responsibility is to integrate recommendations for intervention, monitor progress, and support planning for the future.

#### Provide access to full and effective participation and inclusion in society

The World Health Organisation [90] notes that non-specialist providers in school and community settings can successfully deliver psychosocial interventions, including behavioural approaches and parent-mediated interventions, to children with autism and/or intellectual disability. WHO also states that interventions for autistic people should be accompanied by broader strategies to make physical and social environments more accessible, inclusive, and supportive. Support should be provided within all contexts, including the family home, educational, work and residential settings, local health services, community leisure facilities, as well as local cultural and religious groups, as appropriate.

#### *Respect individual rights*

2016 marked the tenth anniversary of the adoption of the Convention on the United Nations Rights of Persons with Disabilities (CRPD). Remarkable progress has taken place over the last decade and the Convention continues supporting member states in the formulation and enforcement of their laws, strategies, policies, and programmes that promote equality, inclusion, and empowerment of persons with disabilities at global, regional, national, and local levels [91]. These principles have inspired autism organisations in Europe, who urge clinicians to empower people with autism, and their families or carers, to defend their rights, contribute to policy making and participate fully in society.

#### *Establish referral mechanisms and coordinate key agencies*

Throughout the life course of people with autism, various government departments, and local authority or community agencies play a key role. These services traditionally involve health, education, social care, family welfare, housing, employment, taxation, pensions, equal rights legislation, and the justice system more generally. However, many other services also have a significant role, including those involved in scientific research, technology, sports, culture, transportation, and immigration. In other words, people with autism need a multi-dimensional, coordinated action that cuts across sectoral policies at both the national and regional/local level. Unfortunately, in practice, a few countries offer this overarching approach. Nevertheless, as a result of growing awareness of the diversity of autism and its personal and economic costs, many nations in Europe have now approved, or are currently discussing, parliamentary proposals for a comprehensive National Autism Strategy. These include the United Kingdom, Wales, Denmark, Hungary, France, Spain, and Malta as well as other countries across the world.

### Intervention across the lifespan

The support needed for people with autism varies from individual to individual *and* within the same individual over time*.* A recent paper [92] has highlighted the importance of supporting autistic people, across the life span, by maximising potential and minimising barriers to promote inclusion in education, work, and society.

#### Infancy/toddlerhood

It is important that children with autism have access to appropriate intervention and education as early as possible. For this reason, we suggest the following strategies, all of which should take place at more or less the same time.

1. Provide practical advice to parents, educational staff, and all significant adults by means of booklets and other information materials (including relevant Internet sites) that are available in language appropriate for their level of understanding and linguistic and cultural backgrounds. Parents can then be encouraged to disseminate this knowledge among all those involved with the child.
2. Following formal assessment of the child’s developmental level, implement general strategies, based on typical developmental trajectories, to enhance learning and skill acquisition (see, for example, the developmental frameworks described in the Portage Model [93], the Early Start Denver Model [94], PACT [84], the SCERTS model [95], and/or the Parent-Implemented Social Intervention for Toddlers [96].
3. Address the specific social, communication, and behavioural challenges of autism, using interventions based on research and/or expert guidance (See Table S6—examples of current interventions for autism and sections on general Principles above, and specific Interventions below). Individually structured sessions and naturally occurring opportunities throughout the day can be used to enhance joint attention, engagement, and reciprocal communication. With the help of the key worker, supported by other relevant professionals, strategies derived from a variety of intervention models can be modified and combined to develop flexible and individually based intervention programmes that meet the specific needs of the child and family.
4. Consider the child in his or her entirety. Beyond the autism, there is an individual who has the same needs as other children. Pay attention to non-autistic aspects of life, fostering activities that promote general well-being, such as good sleep, healthy nutrition, and exercise.
5. Recognise the value and effort of parents, facilitating access to all relevant support services and providing them with information about local autism societies that now exist throughout most of Europe.

#### School-age period

As children enter the pre-school/elementary school stage, the choice of educational placement becomes a major concern. Different facilities exist throughout Europe, with some countries promoting total inclusion, others offering a mix of mainstream education, and special units attached to mainstream schools and/or specialist schools for children with autism. There is no evidence for the superiority of any single model of schooling, and placement in mainstream school without appropriate support can be highly damaging. The crucial requirement is that every child has access (according to his or her needs) to specialised support staff, autism aware teachers, and a diversity of resources for individual or small group education. In countries where well-supported, inclusive education is not available, the focus should be on building bridges between community and specialist provision. In this way, expert knowledge and experience can be shared across different environments and many more students offered the opportunity to access and profit from inclusive schooling.

For all children with special educational needs, whatever their school placement, adaptations are likely to be required to the general curriculum to ensure that this is appropriate for individual profiles of skills and difficulties. Teaching methods also need to be modified if children are to meet expected educational goals. Modifications may include the use of augmentative communication strategies, greater reliance on visual cues, task analysis, and a high degree of structure of time, environment, and activities. It is also essential that the skills taught will be of value in the years to come, with a particular focus on learning practical daily living skills. And, above all, autistic students must be helped to acquire the social skills that will allow them to become full members of society. Without support, autistic children are unlikely to learn how to socialise successfully with their peers, and peers, too, need help to work, play, and interact successfully with children with autism. Although evidence for any specific social skills training program is limited (see “Specific interventions”), learning to understand, accept, and practice social skills in the context of a real-life school environment is vital for social and academic inclusion.

Teaching staff, in all educational settings, must also be fully aware of the special vulnerabilities of children with autism and, in particular, the high risk of exploitation, bullying, and mistreatment. To decrease the risk of abuse, teachers must focus on ways of empowering all pupils with special needs. They will need to provide their pupils with guidance about identity (including sexual identity and orientation); how to protect their safety on the Internet; how to recognise and report bullying (to themselves or others); and how to enhance self-esteem.

#### Transition to adulthood

Many follow-up studies have highlighted the lack of provision for people with autism, whatever their cognitive level, as they transition from adolescence to adulthood. It is also apparent that individuals of higher cognitive ability are least likely to be offered any specialist support. A few colleges or universities consider their specific needs, despite the fact they may become highly successful students if their academic or career path is tailored to fit with their particular profiles of strengths and interests. Without adequate support, failure to cope with the academic and social demands of university can result in isolation and exclusion and the development of depression and anxiety. However, with support and encouragement, autistic students can become productive members of special-interest groups, play an active role in social media, and/or participate in mutual support groups for individuals with autism. Some also make close friendships and relationships in this way. Supported employment schemes, too, have the potential to help many more autistic adults into worthwhile and well paid employment.

For individuals with autism and intellectual disability, the risk for many is that they are obliged to leave full-time education long before they have acquired the skills required for participation in the community. In turn, this can lead to further social exclusion and rejection. Few have access to individualised, transitional programmes or support for employment or further education, with the result that many remain highly dependent on others, often living with elderly parents. Mental and physical health often deteriorate and challenging behaviours can become more frequent.

#### Adult life

The goal for all adults, whether or not they have autism, is to achieve a quality of life (QoL) that is equivalent to that of the majority of citizens of their country. The World Health Organization (WHO) defines Quality of Life as an individual’s perception of their position in life, in the context of the culture and value systems in which they live and in relation to their goals, expectations, standards, and concerns [97]. There are many different measures of quality of life, and in the field of disabilities, it has been suggested that there are eight essential domains: personal development, self-determination, interpersonal relations, social inclusion, rights, and emotional, physical, and material well-being [98]. The widely utilised WHO measure of quality of life has now been adapted for use with autistic people, following the addition of nine autism-specific items (ASQoL) [99].

Although the evidence base for interventions in adulthood is limited, there is some evidence for interventions designed to improve social functioning [100, 101]. These typically involve a wide range of different strategies including modelling, direct feedback, discussion and decision-making, and formulation of explicit rules and general strategies for dealing with socially difficult situations [59]. For mood disorders, cognitive-behavioural approaches, including mindfulness, are reported to have a moderate-to-large effect on reducing anxiety [102], although the impact on depressive symptoms is less certain [103]. However, adaptations to standard cognitive-behavioural therapy (CBT) are needed to take account of the specific symptoms of autism [104]. Programmes designed to develop recreational, leisure, and daily living skills have also been found to improve mental health and general quality of life [105].

For many autistic adults, however, access to appropriate employment is probably one of the most effective means of improving quality of life. Active participation in the workplace provides a meaningful and structured daily programme, improves social inclusion and cognitive performance, and, financially, increases opportunities for higher quality residential provision and leisure activities. Although individuals with autism face many challenges in finding and retaining suitable employment [106], there is increasing evidence for the effectiveness and cost benefits of supported employment schemes. These take many different forms, but most offer training to develop essential social and work-related skills; advise about modifications to standard employment situations (e.g., adapting interview procedures; making the physical work environment more “autism friendly”, etc.), and provide on-site support for both autistic employees and their employers [107]. These schemes have been found to lead to increased employment rates, higher pay and levels of employment, improved job retention, and reduced dependence on state benefits [108, 109].

Adulthood is the longest period of life and clinicians involved in the transition to adulthood should help to establish a Life Project that includes all the essential shared components of human life. These include support for: independent or semi-independent living; for developing friendships and, as appropriate, intimate relationships; decreasing loneliness and improving community participation, and helping individuals to deal with unavoidable life events such as loss of parents, or the need to move home. It is also essential to develop effective ways of maintaining good physical health and to provide regular medical care and monitoring for chronic conditions associated with autism, particularly epilepsy. Access to autism-specific mental health care is also crucial to reduce the rates of anxiety and depression and minimise the risk of suicide.

To achieve all, this is a challenging agenda, requiring innovative approaches to enhance opportunities for social inclusion and the development of research that truly reflects the priorities of individuals with autism. For example, AUTISTICA, UK has recently worked together with people with autism, professionals, and families to define their Ten Research Priorities, [110]. Other authors [111] also highlight the need for research to be better tailored to community needs and values.

### Specific therapeutic interventions

Despite various claims for “miracle” treatments for autism, there are no specific interventions that can be recommended for all individuals. To date, the strongest evidence, from large-scale randomised control trials, is for interventions that focus on early parent–child interactions. However, most of these have involved very young children and the evidence base for interventions for older children, for adolescents, and particularly adults is limited. Moreover, even in the more successful trials, not all children show significant progress and only a tiny proportion of individuals with autism, or their families, will ever have access to interventions provided by highly skilled researchers and clinicians [46]. Nevertheless, the results from recent reviews of randomised trials and smaller scale group studies [7, 8, 32] suggest a number of general approaches that may be helpful for many children and their families.

#### Developmentally based, social communication therapies

At the present time, the strongest evidence for interventions for young autistic children comes from large-scale, randomised trials of developmentally based approaches designed to facilitate social communication between very young children and their parents. The main focus is on adult–child synchrony, with parents learning to respond to their child’s communicative cues in ways that encourage spontaneous communication, and create opportunities for shared attention, child initiations, and spontaneous play. The most rigorously evaluated of these are the Joint Attention Symbolic Play Engagement and Regulation programme (JASPER) [112] and the Preschool Autism Communication Trial (PACT) [84]. Other programmes with a focus on early parent-mediated interventions have a weaker evidence base and not all trials have produced positive results. These include developmental individual–difference relationship-based model (DIR) or Floortime [113], and Hanen More than Words [114]. Nevertheless, the main principles involved—i.e., parental involvement; using naturalistic opportunities for learning during daily routines; facilitating generalisation of skills across environments—are recommended strategies for improving social communication, especially in very young children [115] (see Supplementary Table S6).

#### Interventions based on applied behaviour analysis

Programmes based primarily on principles of applied behaviour analysis (ABA) have been extensively used in educational and other settings for individuals with autism. The basic strategies of ABA (reinforcement, modelling, prompting, shaping to increase skills, etc.) have been shown, in many single case and small group studies, to result in positive behaviour change [116]. There are also a number of highly manualised and intensive ABA programmes, such as the early intensive behaviour intervention (EIBI) [117], which involves 30–40 h per week of home-based therapy, over 2 years or more. Although EIBI programmes can benefit some children with autism [7], recent reviews highlight the poor quality of much EIBI research and conclude that there is very limited evidence to demonstrate the superiority of intensive ABA programmes [118]. Consequently, many current interventions based on applied behavioural approaches have now shifted from repetitive “discrete trial” training to more natural, child-initiated, developmentally appropriate strategies and tasks [46].

#### Naturalistic developmental behavioural interventions

Recent systematic reviews [119] indicate the potential value of interventions that are based on behavioural strategies and which, although largely adult directed, emphasise the importance of individually based, naturalistic learning. The Early Start Denver Model (ESDM) [86, 94] is probably among the best known of these, with small-scale studies demonstrating improvements in developmental and adaptive behaviours. However, a larger scale trial showed no uniform across-sites advantage over community treatment as usual [120]. Other interventions based on this model with some, albeit weaker supportive evidence, include enhanced milieu teaching (EMT) [121]; incidental teaching (IT) [122], pivotal response treatment (PRT) [123], reciprocal imitation training (RIT) [124], and social communications/emotional regulation/transactional support (SCERTS) [96].

#### Parent focused behavioural management programmes

The intensity and costs of many home or clinic-based behavioural programmes mean that the financial or time demands are far beyond the means of most families, and a few public services are able to provide therapy of this kind. However, it is possible to provide families with evidence-based advice about behavioural management on a much less costly basis. Psycho-education groups for parents of newly diagnosed children are now offered routinely by many child and adolescent clinics and aim to increase parents’ understanding of autism; how to enhance social and communication skills, and how to manage “challenging” behaviours such as rituals, temper tantrums and aggression, fears and phobias, and eating, sleeping, and toileting problems. Programmes are generally group-based (sometimes with some individual and/or home sessions), typically taking place over a few weeks, and have been found to increase parental competence and well-being, and to show improvements in children’s adaptive behaviour [125]. Stepping Stones Triple P is a specific group-based intervention designed to help develop more positive parenting skills; therapists can also offer individual tailored sessions for parents if needed (e.g., for parental mental health issues; child severe behaviour problems). The intervention has been found to be helpful for parents of children with many different types of problems as well as those with autism.[126]. A manualized treatment approach, developed in Germany, applied low-intensity training of parental synchrony and child’s reciprocity and obtained improvement in social communication as well as developmental progress by implementing natural-learning paradigms [127].

#### Social skill programmes

Interventions to improve social skills are widely used in schools and clinics for autistic children. These can involve a wide variety of different strategies, including social groups, computerised programmes, cognitive behaviour strategies, and peer support [128–130]. Most studies report positive findings on parent and/or teacher ratings of social competence, and/or analogue measures of children’s social awareness and understanding. However, evidence of generalisation to “real-life” settings, or to broader groups of autistic children, remains limited. For instance, programmes like the Secret Agent Society (SAS) [131] have shown positive effects, but these are limited to only certain aspects of social skills and to children of average cognitive ability. The effects of computer-based teaching programmes are also limited [132]. Given the variability and complexity of most social skills interventions, and the heterogeneity of participants involved, it is still unclear which are the most effective components [133] or which programmes work best for which children.

#### Other therapies

*Speech and language therapy *(*SLT*), with a focus on increasing comprehension and spontaneous communication has long been an integral part of education for children with autism. There is no strong evidence for the effects of any particular model of SLT and the specific strategies involved very widely from one service to another. The main goal is to enhance understanding and communication by whatever means are most appropriate for the individual, rather than concentrating solely on the production of speech. For many individuals, particularly those of lower ability, this may involve using alternative or augmentative forms of communication, such as signs, symbols, or pictures (e.g., the Picture Exchange Communication System [PECS] [134]. Approaches, such as TEACCH [135] and Social Stories [136], also focus on the importance of using visual cues to enhance comprehension and encourage spontaneous communication among children with limited verbal skills. In addition, TEACCH emphasises the importance of creating an environment that is predictable and as stress free as possible. However, despite being extensively used in schools and other settings for children with autism, the effectiveness of these specific programmes still has to be demonstrated [137]. Recent technological advances also provide greater opportunities for children with autism to communicate using computers, laptops, tablets, and other devices, although over reliance on technology can have disadvantages. For example, in a recent larg-scale randomised control trial of computer-assisted teaching programmes for young autistic children, there was no effect on cognitive or language skills; moreover, pupils who spent most time using the programme were least likely to show improvement [132].

*Occupational therapies*, with a focus on sensory, motor, and adaptive behaviour skills, are also extensively used in many settings to improve daily living skills and adaptive behaviours in individuals with autism. While it is clearly essential to help autistic children minimise motor or sensory difficulties that interfere with daily functioning, few of these interventions have a strong evidence base. In particular, there is little evidence for the effectiveness of sensory integration therapy, which focuses on tactile, vestibular, and/or proprioceptive sensory (e.g., using weighted vests) to support self-regulation [138, 139]. However, a number of single case or small group studies indicate that behaviourally based strategies (e.g., desensitisation, modelling, prompting, and fading) can be used to help children gradually tolerate sensory stimuli that cause distress. Simple environmental modifications (to sound, lighting, clothing, rooms, furniture, etc.) can also significantly reduce problems. There are many strategies, too, that can be used to increase play and improve motor co-ordination, but the specific approaches used should be governed by a careful assessment of each child’s difficulties, skills, likes, and dislikes [140].

In older individuals with autism, there is evidence that better adaptive skills and participation in leisure and recreational activities are significantly associated with lower stress [105]. Hence, occupational therapy for young children should focus on activities such as physical exercise, playing games, and practical daily living skills, which will facilitate social integration in later life.

Although numerous other therapies have been reported, in small or single case studies, to improve cognitive, social, and behavioural functioning in children with autism, these claims are rarely supported by larger scale randomised-controlled trials. For example, while music and animal-based therapies have recently received considerable publicity, the evidence base for these is very weak [141, 142].

#### Interventions for behaviours that challenge

In addition to difficulties associated with the core symptoms of autism, many individuals show the patterns of behaviour that are challenging for themselves and those living or working with them. Such behaviours can have many negative consequences including limiting opportunities for educational, vocational, and social participation; reducing quality of life; constraining personal development, and sometimes causing harm to self-and/or others. Caregivers, too, often experience high levels of stress and poor mental health as a consequence.[143].

UK NICE guidelines [4] recommend that a psychosocial approach to intervention should be the first line of treatment if no co-existing mental or physical disorder has been identified as a possible underlying cause of behavioural disturbance. This approach involves the assessment and modification of environmental factors that contribute to initiating or maintaining the behaviour; the development of a clearly defined intervention strategy that takes into account the individual’s developmental level; systematic measurement to ensure that the agreed outcomes are met within a specified time period, and agreement between the individual, parents and professionals in all settings about how to implement the intervention.

Because “challenging behaviours” can result in very negative and punitive consequences for individuals with autism, recent research has focused on the importance of principles based on positive behaviour support (PBS) [144]. These focus on the teaching of new and more effective skills to facilitate behavioural change and highlight the need to replace coercion with environmental modifications that will result in positive, durable and meaningful change. PBS also emphasises the need for a functional assessment of the possible causes of behavioural difficulties; modification of factors that trigger or maintain the behaviour, and the development of social and communication skills that can replace problem behaviours. Although there are a few randomised control trials of PBS, many single case or small group studies indicate the value of this general approach for reducing severely challenging behaviours and improving the lives of people with autism and those caring for them [145, 146].

#### Treatments for co-occurring conditions

The American Psychiatric Association recently highlighted [147] that over 95% of individuals with autism have at least one additional disorder and many have multiple co-occurring difficulties. These conditions include intellectual disability, sleeping, eating and elimination problems, seizures, gastrointestinal disorders, cerebral palsy, encephalopathy, language problems, visual and hearing impairments, genetic disorders, Tourette syndrome, and, most notably, a wide range of emotional and behavioural problems [53–56].

However, due to symptom overlap, diagnostic overshadowing and atypical symptom presentation, assessment, diagnosis, and treatment of co-occurring conditions in autism are complex and challenging [55]. Although individuals with autism should be provided with appropriate medical treatments for all co-occurring medical conditions, it is increasingly apparent that many experience significant difficulties in accessing medical care [77, 148]. To promote better physical and mental health and reduce the risk of premature death, systems of care must recognise and adapt to the needs of autistic people, as should be done with any vulnerable group in society. In particular, there is a need for joint working relationships between family doctors and clinicians across many different disciplines (paediatrics, neurology, genetics, mental health, intellectual disability, etc.). There is a crucial need, too, for the development of appropriate and valid diagnostic instruments, to identify developmental, physical, sensory, emotional, and behavioural conditions in individuals with autism.

#### Pharmacological treatments

As noted above, emotional and behavioural problems are common in autism and medication may be considered, especially when environmental modifications, or psychosocial or other interventions, based on a detailed functional analysis, prove ineffective. However, medication should be used with a particular caution, especially when treating mental health problems in autism. First, accurate diagnosis presents many challenges. For example, the classical symptoms of depression may present very differently in autism, while anxiety may initially manifest as an increase in behavioural problems. In diagnosing ADHD, it is often difficult to determine whether poor attention is due to a basic deficit in attention/concentration; whether it results from social impairments that affect shared attention with others, or if the topic or activity lie outside the individual’s own special interests. Impulsive behaviours, too, may be influenced by limited awareness of social rules and failure to understand others’ expectations. Similarly, there may be fundamental differences between the anxiety related behaviours that are typically associated with obsessive–compulsive disorder and the compulsions of someone with autism. Thus, repetitive behaviours and restricted interests in autism often tend to be associated with comfort, relief and enjoyment, rather than distress or agitation. Clinicians’ experience and understanding of all these conditions, and how their presentation may differ in autism, are essential to ensure correct diagnosis.

Second, very few drugs have been specifically trialled with children or adults with autism and, indeed, these groups are frequently excluded from clinical trials. Although pharmacological treatments for autism are generally "off-label”, the prescription of such medications for co-existing conditions should follow the professional practice codes established for the general population. In addition, given diagnostic challenges, lack of autism-specific pharmacological trials, and the very often unpredictable response to drugs by autistic people, it is important that medications are prescribed on an individualised and experimental basis. This requires paying careful attention to both positive and negative effects, disseminating information about the particular drug used to key people in the individual’s environment, and conducting systematic and frequent medical follow-ups. Each medication should be started at a low dosage and the duration of treatment should be individually adjusted, depending on the co-occurring condition, the patient, and the documented response. Also, whatever medication is prescribed, it is crucial that any potential benefits are weighed against the risk of side effects.

In the United States of America, two medications, Risperidone and Aripiprazole [149, 150], are approved by the Food and Drug Administration (FDA) for agitation and irritability in autism. Systematic follow-up is required when prescribing either drug, and for Risperidone, in particular, the metabolic side effects must be carefully monitored. In Europe, the European Medicines Agency (EMEA) has approved Haloperidol [151] for persistent, severe aggression in children and adolescents with autism, when other treatments have not worked or cause unacceptable side effects. This, too, is to be used with great caution because of the risk of dyskinetic side effects. A prolonged-release Melatonin product has been recently approved in Europe for the treatment of insomnia in autism (after other psychosocial measures have proved ineffective) [152]. Methylphenidate and guanfacine [153] have been reported to be effective for the management of hyperactivity in some children with autism, although they are used off-label in autism, and many trials suffer from methodological limitations. In the RUPP initial study [154], it was found that short-elimination methylphenidate, although effective, was less beneficial for people with autism than those with ADHD; individuals with autism were also more likely to experience side effects [155]. Guanfacine had similar, moderate effects as methylphenidate for patients with autism (50%), although side effects were not as frequent or serious [156]. Although selective serotonin reuptake inhibitors (SSRIs) are frequently used to treat anxiety and/or compulsions in autism, a Cochrane review and others found little evidence to support their use [157, 158]. If they are used, the risk of behavioural hyperarousal (impulsivity, restlessness, and insomnia) may be reduced by initiating treatment at low doses and if necessary, further decreasing the dosage, or discontinuing the drug [159]. Moreover, when medication is prescribed, this should always be done within a multimodal intervention framework; drug treatments alone, even when indicated, are not a substitute for other treatments [160, 161].

Although current evidence, recently reviewed by the British Association for Psychopharmacology (BAP) [162], discourages the routine use of any pharmacological treatment for the core symptoms of autism, guidance documents such as BAP and NICE [4, 5, 162] provide many recommendations for medical interventions that can be used to treat commonly co-occurring disorders. While standard pharmacological guidelines for these conditions should be followed (for example, melatonin for sleep problems, specific anticonvulsants for epilepsy, dopamine blockers for irritability, methylphenidate, atomoxetine and guanfacines for ADHD, etc.), given the biological and genetic heterogeneity of autism, and the relative lack of autism-specific drug studies, especially for adults, much research on pharmacological treatments is still needed. A 5-year, collaborative program, the AIMS-2-Trials, is currently in progress under the Innovative Medicines Initiative (IMI) of the European Union [163]. This involves 14 countries and 48 academic, charity, and industry partners, together with independent representatives from the autism community. Clinical trials of new compounds have already started, and the programme represents a major effort to place Europe in the forefront of pharmaceutical research in autism. For updates on ongoing clinical trials, readers are referred to the EU Clinical Trials Register [164] and the registry of clinical trials run by the US National Library of Medicine at the National Institutes of Health [165].

### Treatments that should not be used

Despite widespread Internet claims, there is no supportive evidence for diagnostic assays or treatments involving hair analysis, celiac antibodies, allergy testing (particularly food allergies for gluten, casein, *Candida* and other moulds), immunologic or neurochemical abnormalities, micronutrients such as vitamins, intestinal permeability tests, stool analysis, urinary peptides, mitochondrial disorders (including lactate and pyruvate), thyroid function tests, or erythrocyte glutathione peroxidase studies [166]. It is also of paramount importance to make clear that there is NO association between autism and the measles, mumps, and rubella (MMR) vaccine. Misinformation about vaccines and the consequent reluctance of many parents to vaccinate their children is now contributing to outbreaks of measles in parts of the world where the disease had previously been eliminated [167].

There are, at present, no medications to treat the "core" symptoms of autism, and so-called "alternative" treatments (neurofeedback, facilitated communication, auditory integration training, omega-3 fatty acids, secretin, chelation, hyperbaric oxygen therapy, exclusion diets, etc.) have no place in the treatment of core autism features [4, 5].

## Summary and conclusion

On the basis of the research and clinical information reviewed above, this ASD ESCAP Practice Guidance makes the following recommendations:

1. Classification of autism in DSM-5 and in the forthcoming ICD-11 should help to harmonise diagnosis in children and adults. However, diagnosis, of itself, does not establish the kinds of treatment required, and hence, clinical diagnosis should be personalised and contextualised, focusing both on individual limitations and strengths.
2. There are many different measures available for detection, diagnosis, and assessment in autism, and such assessments are essential for identifying individual characteristics and support needs. Nevertheless, there is a need to demonstrate the validity and reliability of these instruments for economically or socially disadvantaged groups, ethnic or racial minorities, or for individuals with co-occurring disorders.
3. Interventions should focus on strategies to make the environment more “autism friendly”, and on understanding the many ways in which we can all support the inclusion of children and adults with autism and their families. This calls for the contribution not only of health, education, and social care, but of all the community services that are important for a good quality of life.
4. Instead of viewing themselves as “exclusive experts” professionals should focus on becoming the coaches of those individuals who love, live, work, and/or care for autistic people. They are the ones who will have a positive and lasting impact on the successful development of the person, and on his or her quality of life.
5. Because so few interventions for autism have been tested in large-scale randomised control trials, it is not possible to rely solely on such evidence to provide autistic people with treatment and support. Intervention should be based on scientific, empirical evidence, but, given the limitations of current research trials, recommendations for general treatment strategies must also consider guidance from teams of international experts; be compatible with societal values, and have the endorsement of the person with autism and/or his or her legal guardians.
6. We fully acknowledge and applaud the outstanding accomplishments of those individuals for whom autism may be a benefit, even a gift. Nevertheless, as practitioners, we have a duty to direct our efforts towards those who are disadvantaged by autism; the many millions of citizens across Europe and the world whose human rights, including medical and psychiatric needs, continue to be unmet.

## Electronic supplementary material

Below is the link to the electronic supplementary material.

Supplementary file1 (DOCX 56 kb)

## Footnotes

1 In this document, we use a combination of “person first” language (e.g., “people with autism”) as well as “identity first” language (e.g., “autistic people”) to reflect the different views on terminology among those in the autism community, parents and practitioners [12].

2 Interested readers can review the degree of evidence classification of diverse treatments in guidelines freely available on the Internet, such as those from the American Academy of Child and Adolescent Psychiatry, Australia. Belgium, California, France, Germany, India, Italy, Manchester/Maine, Missouri, New York City, Ontario, Sidney, Spain, Scotland, UK (NICE Guidelines), and New Zealand, among other regions of the world.

## References

[1] Díez-Cuervo A, Muñoz-Yunta JA, Fuentes-Biggi J, Canal-Bedia R, Idiazábal-Aletxa MA (2005). Guía de buena práctica para el diagnóstico de los trastornos del espectro autista. Grupo de Estudio de los Trastornos del Espectro Autista del Instituto de Salud Carlos III [Article in Spanish]. Rev Neurol.

[2] Fuentes-Biggi J, Ferrari-Arroyo MJ, Boada-Muñoz L, Touriño-Aguilera E, Artigas-Pallarés J (2006). Guía de buena práctica para el tratamiento de los trastornos del espectro autista. Grupo de Estudio de los Trastornos del Espectro Autista del Instituto de Salud Carlos III [Article in Spanish]. Rev Neurol.

[3] National Institute for Health and Care Excellence (NICE) (2011) Autism spectrum disorder in under 19s: recognition, referral and diagnosis. https://www.nice.org.uk/guidance/cg128. Accessed 5 May 2020 31999412

[4] National Institute for Health and Care Excellence (NICE) (2013) Autism spectrum disorder in under 19s: support and management. https://www.nice.org.uk/guidance/cg170. Accessed 5 May 2020 34283415

[5] National Institute for Health and Care Excellence (NICE) (2012) Autism spectrum disorder in adults: diagnosis and management. https://www.nice.org.uk/guidance/cg142 . Accessed 5 May 2020 32186834

[6] Volkmar F, Siegel M, Woodbury-Smith M, King B, McCracken J, State M, American Academy of Child and Adolescent Psychiatry (AACAP) Committee on Quality Issues (CQI) (2014). Practice parameter for the assessment and treatment of children and adolescents with autism spectrum disorder. J Am Acad Child Adolesc Psychiatry.

[7] New York State Department of Health Clinical Practice Guideline on Assessment and Intervention Services for Young Children (Age 0–3) with Autism Spectrum Disorders (ASD): 2017 Update. New York State Department of Health Bureau of Early Intervention. https://www.health.ny.gov/community/infants_children/early_intervention/autism/docs/report_recommendations_update.pdf. Accessed 5 May 2020

[8] Whitehouse AJO, Evans K, Eapen V, Wray J (2018). A national guideline for the assessment and diagnosis of autism spectrum disorders in Australia.

[9] Scottish Intercollegiate Guidelines Network (2016) Assessment, diagnosis and interventions for autism spectrum disorders. (SIGN publication no. 145—revalidated in 2019) https://www.sign.ac.uk/sign-145-assessment,-diagnosis-and-interventions-for-autism-spectrum-disorders.html. Accessed 5 May 2020

[10] American Psychiatric Association (2013). Diagnostic and Statistical Manual of Mental Disorders.

[11] Misés R, Bursztein C, Botbol M, Coinçon Y, Durand B (2012). Une nouvelle version de la classification française des troubles mentaux de l’enfant et de l’adolescent : la CFTMEA R 2012, correspondances et transcodages avec l’ICD 10. Neuropsychiatrie de l’enfance et de la Adelescence.

[12] Kenny L, Hattersley C, Molins B, Buckley C, Povey C, Pellicano E (2016). Which terms should be used to describe autism? Perspectives from the UK autism community. Autism.

[13] American Psychiatric Association (2000). Diagnostic and statistical manual of mental disorders.

[14] World Health Organization (1992). The ICD-10 classification of mental and behavioural disorders: clinical descriptions and diagnostic guidelines.

[15] Buxbaum JD, Baron-Cohen S (2013). DSM-5: the debate continues. Mol Autism.

[16] Volkmar FR, McPartland JC (2014). From Kanner to DSM-5: autism as an evolving diagnostic concept. Annu Rev Clin Psychol.

[17] World Health Organization (2018) International statistical classification of diseases and related health problems (11th Revision). https://icd.who.int/browse11/l-m/en. Accessed 5 May 2020

[18] WHO Autism Spectrum Disorders (2019) https://www.who.int/news-room/fact-sheets/detail/autism-spectrum-disorders. Accessed 5 May 2020

[19] Fombonne E (2018). Editorial: the rising prevalence of autism. J Child Psychol Psychiatry.

[20] Elsabbagh M, Divan G, Koh YJ, Kauchali S, Marcín C, Montiel-Nava C (2012). Global prevalence of autism and other pervasive developmental disorders. Autism Res.

[21] Maenmer MJ, Shaw KA, Baio J, Washington A, Patrick M, et al. Prevalence of autism spectrum disorder among children aged 8 years—autism and developmental disabilities monitoring network, 11 sites, United States, 2016. MMRW Surveill Summ (2020); 69 (No. SS-4): 1–12. https://www.cdc.gov/mmwr/volumes/69/ss/ss6904a1.htm. Accessed 5 May 2020 10.15585/mmwr.ss6904a1 PMC7119644 32214087

[22] Christensen DL, Maenner MJ, Bilder D, Constantino JN, Daniels J (2019). Prevalence and characteristics of autism spectrum disorder among children aged 4 years—early autism and developmental disabilities monitoring network, seven sites, United States, 2010, 2012, and 2014. MMWR Surveill Summ.

[23] Daniels AM, Mandell DS (2014). Explaining differences in age at autism spectrum disorder diagnosis: a critical review. Autism.

[24] Richards M, Mossey J, Robins DL (2016). Parents’ concerns as they relate to their child’s development and later diagnosis of autism spectrum disorder. J Dev Behav Pediatr JDBP.

[25] Becerra-Culqui TA, Linch FL, Owen-Smith AA, Spitzer J, Croen LA (2018). Parental first concerns and timing of autism spectrum disorder diagnosis. J Autism Dev Disord.

[26] National Center on Birth Defects and Developmental Disabilities (2019) Centers for disease control and prevention: signs and symptoms of autism spectrum disorders: possible "red flags". https://www.cdc.gov/ncbddd/autism/signs.html. Accessed 5 May 2020

[27] CNN Health + (2008) Vaccine case draws new attention to autism debate. https://edition.cnn.com/2008/HEALTH/conditions/03/06/vaccines.autism/index.html. Accessed 5 May 2020

[28] Baird G, Charman T, Pickles A, Chandler S, Loucas T (2008). Regression, developmental trajectory and associated problems in disorders in the autism spectrum: the SNAP study. J Autism Dev Disord.

[29] Mantovani JF (2000). Autistic regression and Landau-Kleffner syndrome: progress or confusion?. Dev Med Child Neurol.

[30] Rogers SJ (2004). Developmental regression in autism spectrum disorders. Ment Retard Dev Disabil Res Rev.

[31] Ozonoff S, Iosif AM (2019). Changing conceptualizations of regression: what prospective studies reveal about the onset of autism spectrum disorder. Neurosci Biobehav Rev.

[32] Hyman SL, Levy SE, Myers SC (2020). Identification, evaluation, and management of children with autism spectrum disorder. Pediatrics.

[33] Siu AL, Bibbins-Domingo K, Grossman DC, Baumann LC, Davidson KW (2016). Screening for autism spectrum disorder in young children: US preventive services task force recommendation statement. JAMA.

[34] Modabbernia A, Velthorst E, Reichenberg A (2017). Environmental risk factors for autism: an evidence-based review of systematic reviews and meta-analyses. Mol Autism.

[35] Richards C, Jones C, Groves L, Moss J, Oliver C (2015). Prevalence of autism spectrum disorder phenomenology in genetic disorders: a systematic review and meta-analysis. Lancet Psychiatry.

[36] Towbin K, Pradella A, Gorrindo T, Pine D, Leibenluft E (2005). Autism Spectrum traits in children with mood and anxiety disorders. J Child Adolesc Psychopharmacol.

[37] Leitner Y (2014). The co-occurrence of autism and attention deficit hyperactivity disorder in children—what do we know?. Front Hum Neurosci.

[38] Stewart E, Cancilliere MK, Freeman J, Wellen B, Garcia A (2016). Elevated autism spectrum disorder traits in young children with OCD. Child Psychiatry Hum Dev.

[39] Zuckerman KE, Lindley OJ, Sinche BK (2015). Parental concerns, provider response, and timeliness of autism spectrum disorder diagnosis. J Pediatr.

[40] Jobs EN, Bölte S, Falck-Ytter T (2019). Spotting signs of autism in 3-year-olds: comparing information from parents and preschool staff. J Autism Dev Disord.

[41] Green J, Pickles A, Pasco G, Bedford R, Wan MW (2017). Randomised trial of a parent-mediated intervention for infants at high risk for autism: longitudinal outcomes to age 3 years. J Child Psychol Psychiatry.

[42] Dawson G, Rogers S, Munson J, Smith M, Winter J (2010). Randomized controlled trial of an intervention for toddlers with autism: the early start denver model. Pediatrics.

[43] Levy SE, Wolfe A, Coury D (2020). Screening tools for autism spectrum disorders in primary care: a systematic evidence review. Pediatrics.

[44] Vllasaliu L, Jensen K, Dose M, Hagenah U, Hollman H (2019). The diagnostics of autism spectrum disorder in children, adolescents and adults: overview of the key questions and main results of the first part of the German AWMF—Clinical Guideline [Article in German]. Z Kinder Jugendpsychiatr Psychother.

[45] Ecker C (2017). The neuroanatomy of autism spectrum disorder: an overview of structural neuroimaging findings and their translatability to the clinical setting. Autism.

[46] Lord C, Brugha TS, Charman T, Cusack J, Dumas TF (2020). Autism spectrum disorder. Nat Rev Dis Primers.

[47] National Institute of Neurological Disorders and Stroke (2019) Rett Syndrome Fact Sheet. https://www.ninds.nih.gov/Disorders/Patient-Caregiver-Education/Fact-Sheets/Rett-Syndrome-Fact-Sheet. Accessed 5 May 2020

[48] Schaefer GB, Mendelsohn NJ, Practice P, Committee G (2013). Clinical genetics evaluation in identifying the etiology of autism spectrum disorders: 2013 guideline revisions. Genet Med.

[49] Griesi-Oliveira K, Laurato Sertié A (2017). Autism spectrum disorders: an updated guide for genetic counseling. Einstein (Sao Paulo).

[50] Lord C, Elsabbagh M, Baird G, Veenstra-Vanderweele J (2018). Autism spectrum disorder. Lancet.

[51] Sullivan PF, Geschwind DH (2019). Defining the genetic, genomic, cellular, and diagnostic architectures of psychiatric disorders. Cell.

[52] Satterstrom FK (2020). Large-scale exome sequencing study implicates both developmental and functional changes in the neurobiology of autism. Cell.

[53] Lai MC, Kassee C, Besney R, Bonato S, Hull L (2019). Prevalence of co-occurring mental health diagnoses in the autism population: a systematic review and meta-analysis. Lancet Psychiatry.

[54] Darrow SM, Grados M, Sandor P, Hirschtritt ME, Illmann C (2017). Autism spectrum symptoms in a Tourette’s disorder sample. J Am Acad Child Adolesc Psychiatry.

[55] Rosen TE, Mazefsky CA, Vasa RA, Lerner MD (2018). Co-occurring psychiatric conditions in autism spectrum disorder. Int Rev Psychiatry.

[56] Soke GN, Maenner MJ, Christensen D, Kurzus-Spencer M, Schieve LA (2018). Prevalence of co-occurring medical and behavioral conditions/symptoms among 4- and 8-year-old children with autism spectrum disorder in selected areas of the United States in 2010. J Autism Dev Disord.

[57] King RA, The AACAP Work Group on Quality Issues (1997). Practice parameters for the psychiatric assessment of children and adolescents. J Am Acad Child Adolesc Psychiatry.

[58] McGuire K, Fung LK, Hagopian L, Vasa RA, Mahajan R (2016). Irritability and problem behavior in autism spectrum disorder: a practice pathway for pediatric primary care. Pediatrics.

[59] Autism Speaks (2018) Autism speaks challenging behavior tool kit. https://www.autismspeaks.org/sites/default/files/201808/Challenging%2520Behaviors%2520Tool%2520Kit.pdf. Accessed 5 May 2020

[60] Lai MC, Baron-Cohen S (2015). Identifying the lost generation of adults with autism spectrum conditions. Lancet Psychiatry.

[61] Loomes R, Hull L, Mandy WPL (2017). What is the male-to-female ratio in autism spectrum disorder? A systematic review and meta-analysis. J Am Acad Child Adolesc Psychiatry.

[62] Brugha TS, MacManus S, Bankart J, Scott F, Purdon S (2011). Epidemiology of autism spectrum disorders in adults in the community in England. Arch Gen Psychiatry.

[63] Lai MC, Szatmari P (2020). Sex and gender impacts on the behavioural presentation and recognition of autism. Curr Opin Psychaiatry.

[64] Dean M, Harwood R, Kasari C (2017). The art of camouflage: gender differences in the social behaviors of girls and boys with autism spectrum disorder. Autism.

[65] Lai MC, Lombardo MV, Ruigrok AN, Chakrabarti B, Auyeung B (2017). Quantifying and exploring camouflaging in men and women with autism. Autism.

[66] Ratto AB, Kenworthy L, Yeris BE, Bascom J, Wieckowski AT (2018). What about the girls? Sex-based differences in autistic traits and adaptive skills. J Autism Dev Disord.

[67] Nielsen S, Anckarsäter H, Gillberg C, Gillberg C, Rastam M, Wentz E (2015). Effects of autism spectrum disorders on outcome in teenage-onset anorexia nervosa evaluated by the Morgan-Russel outcome assessment schedule: a controlled community-based study. Mol Autism.

[68] Green RM, Travers AM, Howe Y, McDougle CJ (2019). Women and autism spectrum disorder: diagnosis and implications. Curr Psychiatry Rep.

[69] Moss J, Howlin P (2009). Autism spectrum disorders in genetic syndromes: implications for diagnosis, intervention and understanding the wider autism spectrum disorder population. J Intellectual Disabil Res.

[70] Pickles A, McCauley JB, Pepa LA, Huerta M, Lord C (2020). The adult outcome of children referred for autism: typology and prediction from childhood. J Child Psychol Psychiatry.

[71] Simonoff E, Kent R, Stringer D, Lord C, Briskman J (2019). Trajectories in symptoms of autism and cognitive ability in autism from childhood to adult life: findings from a longitudinal epidemiological cohort. J Am Acad Child Adolesc Psychiatry.

[72] Orinstein AJ, Suh J, Porter K, De Yoe KA, Tyson KE (2015). Social function and communication in optimal outcome children and adolescents with an autism history on structured test measures. J Autism Dev Disord.

[73] Howlin P, Magiati I (2017). Autism spectrum disorder: outcomes in adulthood. Curr Opin Psychiatry.

[74] Croen LA, Zerbo O, Qian Y, Massolo ML, Rich S (2015). The health status of adults on the autism spectrum. Autism.

[75] Cashin A, Buckley T, Trollor JN, Lennox N (2018). A scoping review of what is known of the physical health of adults with autism spectrum disorders. J Intellectual Disabil.

[76] Hirvikoski T, Mittendorfer-Rutz E, Boman M, Larsson H, Lichtenstein O, Bölte S (2016). Premature mortality in autism spectrum disorder. Br J Psychaitry.

[77] Mason D, Ingham B, Urbanowicz A, Heather M, Michael C, Birtles H (2019). A systematic review of what barriers and facilitators prevent and enable physical healthcare services access for autistic adults. J Autism Dev Disord.

[78] Smith DaWalt L, Hong J, Greenberg JS, Mailick MR (2019). Mortality in individuals with autism spectrum disorder: predictors over a 20-year period. Autism.

[79] Roestorf A (2018) Ageing, cognition and quality of life in autism spectrum disorder: cross-sectional and longitudinal studies. https://www.researchgate.net/publication/333699846_Ageing_cognition_and_quality_of_life_in_autism_spectrum_disorder_cross-sectional_and_longitudinal_studies. Accessed 5 May 2020

[80] Barnard-Brak L, Richman D, Zhanxia Y (2019). Age at death and comorbidity of dementia-related disorders among individuals with autism spectrum disorder. Adv Autism Emerald Insight.

[81] Matasaka N (2017). Implications of the idea of neurodiversity for understanding the origins of developmental disorders. Phys Life Rev.

[82] Constantino JN, Todd RD (2003). Autistic traits in the general population: a twin study. Arch Gen Psychiatry.

[83] Morales-Hidalgo P, Roigé-Castellví J, Hernández-Martínez C, Voltas N, Canals J (2018). Prevalence and characteristics of autism spectrum disorders among Spanish school-age children. J Autism Dev Disord.

[84] Pickles A, Le Couteur A, Leadbitter K, Salomone E, Cole-Fletcher R (2016). Parent-mediated social communication therapy for young children with autism (PACT): long-term follow-up of a randomised controlled trial. Lancet.

[85] Kasari C, Gulsrud A, Freeman S, Paparella T, Hellemann G (2012). Longitudinal follow-up of children with autism receiving targeted interventions on joint attention and play. J Am Acad Child Adolesc Psychiatry.

[86] Rogers SJ, Estes A, Vismara L, Munson J, Zierhut C (2019). Enhancing low-intensity coaching in parent implemented Early Start Denver Model intervention for early autism: a randomized comparison treatment trial. J Autism Dev Disord.

[87] Divan G, Vajaratkar V, Cardozo P, Huzurbazar S, Verma M (2019). The Feasibility and Effectiveness of PASS Plus: a lay health worker delivered comprehensive intervention for Autism Spectrum Disorders: pilot RCT in a rural low and middle income country setting. Autism Res.

[88] Sandbank M, Bottema-Beutel K, Crowley S, Cassidy M, Dunham K (2020). Project AIM: Autism intervention meta-analysis for studies of young children. Psychol Bull.

[89] Abubakar A, Ssewanyana D, Newton CR (2016). A systematic review of research on Autism Spectrum Disorders in sub-Saharan Africa. Behav Neurol.

[90] World Health Organization (2013) Meeting report: autism spectrum disorders and other developmental disorders: from raising awareness to building capacity: World Health Organization. Geneva, Switzerland. https://apps.who.int/iris/handle/10665/103312. Accessed 5 May 2020

[91] United Nations—Department of Economic and Social Affairs (2016) 10th anniversary of the adoption of Convention on the Rights of Persons with Disabilities (CRPD). https://www.un.org/development/desa/disabilities/convention-on-the-rights-of-persons-with-disabilities/the-10th-anniversary-of-the-adoption-of-convention-on-the-rights-of-persons-with-disabilities-crpd-crpd-10.html. Accessed 5 May 2020

[92] Lai MC, Anagnostou E, Wiznitzer M, Allison C, Baron-Cohen S (2020). Evidence-based support for autistic people across the lifespan: maximising potential, minimising barriers, and optimizing the person-environment fit. Lancet Neurol.

[93] Cooperative Educational Service Agency 5, Portage, WI. (2019) Portage System 3. https://sites.google.com/cesa5.org/portageproject/products/portage-guide-3. Accessed 5 May 2020

[94] Rogers S (2016). Early start denver model. Comprehensive models of autism spectrum disorder treatment.

[95] Prizant B, Wetherby A, Rubin E, Laurent A, Rydell P (2006). The SCERTS model: a comprehensive educational approach for children with autism spectrum disorders.

[96] Wetherby AM, Guthrie W, Woods J, Schatschneider C, Holland R (2014). Parent-implemented social intervention for toddlers with autism: an RCT. Pediatrics.

[97] World Health Organization - Health statistics and information systems. WHOQOL: Measuring Quality of Life. https://www.who.int/healthinfo/survey/whoqol-qualityoflife/en. Accessed 5 May 2020

[98] Schalock RL, Verdugo MA, Gomez LE, Reinders HS (2016). Moving us toward a theory of individual quality of life. Am J Intellectual Dev Disabil.

[99] McConachie H, Mason D, Parr JR, Garland D, Wilson C, Rodgers J (2018). Enhancing the validity of a quality of life measure for autistic people. J Autism Dev Disord.

[100] Ke F, Whalon K, Yun J (2018). Social skill interventions for youth and adults with autism spectrum disorder: a systematic review. Rev Educ Res.

[101] Lorenc T, Rodgers M, Marshall D, Melton H, Rees R (2018). Support for adults with autism spectrum disorder without intellectual impairment: systematic review. Autism.

[102] Sizoo B, Kuiper E (2017). Cognitive behavioural therapy and mindfulness based stress reduction may be equally effective in reducing anxiety and depression in adults with autism spectrum disorders. Res Dev Disabil.

[103] White SW, Simmons GL, Gotham KO, Conner CM, Smith IC (2018). Psychosocial treatments targeting anxiety and depression in adolescents and adults on the autism spectrum: review of the latest research and recommended future directions. Curr Psychiatry Rep.

[104] Spain D, Sin J, Harwood L, Mendez MA, Happé F (2017). Cognitive behaviour therapy for social anxiety in autism spectrum disorder: a systematic review. Adv Autism.

[105] Bishop-Fitzpatrick L, Smith DaWalt L, Greenberg JS, Mailick MR (2017). Participation in recreational activities buffers the impact of perceived stress on quality of life in adults with autism spectrum disorder. Autism Res.

[106] Harmuth E, Silletta E, Bailey A, Adams T, Beck C, Barbic S (2018). Barriers and facilitators to employment for adults with autism: a scoping review. Ann Int Occup Therapy.

[107] Hedley D, Uljarević M, Cameron L, Halder S, Richdale A, Dissanayake C (2017). Employment programmes and interventions targeting adults with autism spectrum disorder: a systematic review of the literature. Autism.

[108] Mavranezouli I, Megnin-Viggars O, Cheema N, Howlin P, Baron-Cohen S, Pilling S (2014). The cost-effectiveness of supported employment for adults with autism in the United Kingdom. Autism.

[109] Wehman P, Schall C, McDonough J, Simma A, Brooke W (2019). Competitive employment for transition-aged youth with significant impact from autism: a multi-site randomized clinical trial. J Autism Dev Disord.

[110] Cusack J, Sterry R (2016) Autistica: your questions shaping future autism research. https://www.autistica.org.uk/downloads/files/Autism-Top-10-Your-Priorities-for-Autism-Research.pdf. Accessed 5 May 2020

[111] Hudry K, Pellicano E, Uljarević M, Whitehouse A (2020). Setting the research agenda to secure the wellbeing of autistic people. Lancet Neurol.

[112] Kasari C, Freeman S, Paparella T (2006). Joint attention and symbolic play in young children with autism: a randomized controlled intervention study. J Child Psychol Psychiatry.

[113] The Interdisciplinary Council on Development and Learning, Inc. DIR, Floortime, and the DIRFloortime (2018) https://www.icdl.com/home. Accessed 5 May 2020

[114] More Than Words—the Hanen Program for Parents of Children with autism spectrum disorder or social communication difficulties (2016) https://www.hanen.org/Programs/For-Parents/More-Than-Words.aspx. Accessed 5 May 2020

[115] Zwaigenbaum L (2015). Early intervention for children with autism spectrum disorder under 3 years of age: recommendations for practice and research. Pediatrics.

[116] Schreibman L (2000). Intensive behavioral/psychoeducational treatments for autism: research needs and future directions. J Autism Dev Disord.

[117] Lovaas OI (1987). Behavioral treatment and normal educational and intellectual functioning in young autistic children. J Consult Clin Psychol.

[118] Reichow B, Hume K, Barton EE, Boyd BA (2018). Early intensive behavioral intervention (EIBI) for young children with autism spectrum disorders (ASD). Cochrane Database Syst Rev.

[119] Tiede G, Walton KM (2019). Meta-analysis of naturalistic developmental behavioral interventions for young children with autism spectrum disorder. Autism.

[120] Rogers S (2019). A multisite randomized controlled two-phase trial of the early start denver model compared to treatment as usual. J Am Acad Child Adolesc Psychiatry.

[121] Hancock TB, Ledbetter-Cho K, Howell A, Lang R (2016). Enhanced milieu teaching. Early intervention for young children with autism spectrum disorder.

[122] McGee GG, Morrier MJ, Daly T (1999). An incidental teaching approach to early intervention for toddlers with autism. J Assoc Persons Severe Handicaps.

[123] Ona HN, Larsen K, Nordheim LV et al (2020) Effects of pivotal response treatment (PRT) for children with autism spectrum disorders (ASD): a systematic review. Rev J Autism Dev Disord 7:78–90. 10.1007/s40489-019-00180-z

[124] Ingersoll B, Schreibman L (2006). Teaching reciprocal imitation skills to young children with autism using a naturalistic behavioral approach: effects on language, pretend play, and joint attention. J Autism Dev Disord.

[125] Tonge B, Brereton A, Kiomall M, Mackinnon A, Rinehart NJ (2014). A randomised group comparison controlled trial of ‘preschoolers with autism’: a parent education and skills training intervention for young children with autistic disorder. Autism.

[126] Kasperzack D, Schrott B, Mingebach T, Becker K, Burghardt R, Kamp-Becker I (2019). Effectiveness of the stepping stones triple P group parenting program in reducing comorbid behavioral problems in children with autism. Autism.

[127] Freitag CM, Jensen K, Teufel K (2020). Empirisch untersuchte entwicklungsorientierte und verhaltenstherapeutisch basierte Therapieprogramme zur Verbesserung der Kernsymptome und der Sprachentwicklung bei Klein- und Vorschulkindern mit Autismus-Spektrum-Störungen [Empirically based developmental and behavioral intervention programs targeting the core symptoms and language development in toddlers and preschool children with autism spectrum disorder] [Article in German]. Z Kinder Jugendpsychiatr Psychother.

[128] Laugeson EA, Frankel F, Gantman A, Dillon AR, Mogil C (2012). Evidence-based social skills training for adolescents with autism spectrum disorders: the UCLA PEERS program. J Autism Dev Disord.

[129] Einfeld SL, Beaumont R, Clark T, Clarke KS, Costley D, Gray KM (2018). School-based social skills training for young people with autism spectrum disorders. J Intellectual Dev Disabil.

[130] Koning C, Magill J, Volden J, Dick B (2013). Efficacy of cognitive behavior therapy-based social skills intervention for school-aged boys with autism spectrum disorders. Res Autism Spectrum Disord.

[131] Secret Agent Society (2020). https://www.sst-institute.net. Accessed 5 May 2020

[132] Pellecchia M, Marcus SC, Spaulding C, Seidman M, Xie M (2020). Randomized trial of a computer-assisted intervention for children with autism in schools. J Am Acad Child Adolesc Psychiatry.

[133] Gates JA, Kang E, Lerner MD (2017). Efficacy of group social skills interventions for youth with autism spectrum disorder: a systematic review and meta-analysis. Clin Psychol Rev.

[134] Flippin M, Reszka S, Watson LR (2010). Effectiveness of the Picture Exchange Communication System (PECS) on communication and speech for children with autism spectrum disorders: a meta-analysis. Am J Speech Lang Pathol.

[135] The University of North Carolina TEACCH Autism Program (2020) https://teacch.com. Accessed 5 May 2020

[136] Karal MA, Wolfe PS (2018). Social story effectiveness on social interaction for students with autism: a review of the literature. Educ Train Autism Dev Disabil.

[137] Sanz-Cervera P, Fernández-Andrés I, Pastor-Cerezuela G, Tárraga-Mínguez R (2018). The effectiveness of TEACCH intervention in autism spectrum disorder: a review study. Papeles Del Psicólogo.

[138] Case-Smith J, Weaver L, Fristad M (2014). A systematic review of sensory processing interventions for children with autism spectrum disorders. Autism.

[139] Lang R, O’Reilly M, Healy O, Rispoli M, Lydon H (2012). Sensory integration therapy for autism spectrum disorders: a systematic review. Res Autism Spectrum Disord.

[140] Case-Smith J, Arbesman M (2008). Evidence-based review of interventions for autism used in or relevance to occupational therapy. Am J Occup Ther.

[141] James R, Sigafoos J, Green VA, Lancioni GE, O’Really MF (2015). Music therapy for individuals with autism spectrum disorder: a systematic review. Rev J Autism Dev.

[142] Maber-Aleksandrowicz S, Avent C, Hassiotis A (2016). A systematic review of animal-assisted therapy on psychosocial outcomes in people with intellectual disability. Res Dev Disabil.

[143] Davis TN, Rispoli M (2018). Introduction to the Special Issue: interventions to reduce challenging behavior among individuals with autism spectrum disorder. Behav Modifi.

[144] McLaughlin DM, Smith CE (2017). Positive behavior support. Handbook of treatments for autism spectrum disorder.

[145] Lavigna G, Willis TJ (2012). The efficacy of positive behavioural support with the most challenging behaviour: the evidence and its implications. J Intellect Dev Disabil.

[146] MacDonald A, McGill P (2013). Outcomes of staff training in positive behaviour support: a systematic review. J Dev Phys Disabil.

[147] American Psychiatric Association (2020) Autism often accompanied by other conditions. https://www.psychiatry.org/news-room/apa-blogs/apa-blog/2018/10/autism-often-accompanied-by-other-conditions. Accessed 5 May 2020

[148] Zerbo O, Qian Y, Ray T, Sidney S, Rich S (2019). Health Care service utilization and cost among adults with autism spectrum disorders in a U.S. integrated health care system. Autism Adulthood.

[149] Drugs@FDA: FDA approved drug products: Risperidone (2006). https://www.accessdata.fda.gov/drugsatfda_docs/label/2019/020272s082,020588s070,021444s056lbl.pdf. Accessed 5 May 2020

[150] Drugs@FDA: FDA Approved Drug Products: Aripiprazole (2009). https://www.accessdata.fda.gov/scripts/cder/daf/index.cfm?event=overview.process&ApplNo=021436. Accessed 5 May 2020

[151] European Medicines Agency: Haloperidol (2017). https://www.ema.europa.eu/en/medicines/human/referrals/haldol-associated-names. Accessed 5 May 2020

[152] European Medicines Agency: Slenyto (2018). https://www.ema.europa.eu/en/medicines/human/EPAR/slenyto#authorisation-details-section. Accessed 5 May 2020

[153] Politte LC, Scahill L, Figueroa J, McCracken JT, King B, McDougle CJ (2018). A randomized, placebo-controlled trial of extended-release guanfacine in children with autism sctrum disorder and ADHD symptoms: an analysis of secondary outcome measures. Neuropsychopharmacology.

[154] Research Units on Pediatric Psychoparmacology Autism Network (2005). Randomized, controlled, crossover trial of methylphenidate in pervasive developmental disorders with hyperactivity. Arch Gen Psychiatry.

[155] Sturman N, Deckx L, van Driel ML (2017). Methylphenidate for children and adolescents with autism spectrum disorder. Cochrane Database Syst Rev.

[156] Scahill L, McCracken JT, King BH, Rockhill C, Shan B, Politte L (2015). Extended-release guanfacine for hyperactivity in children with autism spectrum disorder. Am J Psychiatry.

[157] Williams K, Brignelli A, Randall M, Silove N, Hazell P (2013). Selective serotonin reuptake inhibitors (SSRIs) for autism spectrum disorders (ASD). Cochrane Database Syst Rev.

[158] Goel R, Hong JS, Findling RL, Ji NY (2018). An update on pharmacotherapy of autism spectrum disorder in children and adolescents. Int Rev Psychiatry.

[159] Luft MJ, Lamy M, DelBello MP, McNamara RK, Strawn JR (2018). Antidepressant-induced activation in children and adolescents: risk, recognition and management. Curr Problems Pediatric Adolesc Health Care.

[160] Scahill L, McDougle CJ, Aman MG, Johnson C, Handen B (2012). Effects of risperidone and parent training on adaptive functioning in children with pervasive developmental disorders and serious behavioral problems. J Am Acad Child Adolesc Psychiatry.

[161] Handen BL, Aman MG, Arnold LE, Hyman SL, Tumuluru RV (2015). Atomoxetine, parent training and their combination in children with autism spectrum disorders and attention-deficit/hyperactivity disorder. J Am Acad Child Adolesc Psychiatry.

[162] Howes OD, Rogdaki M, Findon JL, Wichers RH, Charman T (2018). Autism spectrum disorder: consensus guidelines on assessment, treatment and research from the British Association for Psychopharmacology. J Psychopharmacol.

[163] Autism Innovative Medicine Studies-2-Trials (AIMS-2-TRIALS) (2020). https://www.aims-2-trials.eu. Accessed June 2020

[164] EU Clinical Trials Register (2020). https://www.clinicaltrialsregister.eu/ctr-search/search. Accessed 5 May 2020

[165] ClinicalTrials.gov: a resource provided by the U.S. National Library of Medicine (2020). https://clinicaltrials.gov. Accessed 5 May 2020

[166] Centers for Diseases Control and Prevention. Autism Spectrum Disorder. Recommendations and Guidelines (2020). https://www.cdc.gov/ncbddd/autism/hcp-recommendations.html. Accessed 5 May 2020

[167] Fombonne E, Goin-Kochel RP, O’Roak BJ, Abbeduto L, Aberbach G (2020). Beliefs in vaccine as causes of autism among SPARK cohort caregivers. Vaccine.

