# Supplementary material for: ESCAP practice guidance for autism: a summary of evidence-based recommendations for diagnosis and treatment
Source: Eur Child Adolesc Psychiatry. 2020 Jul 14;30(6):961–84. doi: 10.1007/s00787-020-01587-4 (PMC8140956; doi:10.1007/s00787-020-01587-4)
Supplement: Supplementary file 1 — Supplementary file1 (DOCX 56 kb) [file 787_2020_1587_MOESM1_ESM.docx]

SUPPLEMENTARY MATERIAL

*Table S1 - Examples of frequently used screening questionnaires for young children*

| *Scale* | *Informant* | *Age range* | *Number of items* | *Time to administer* |
| --- | --- | --- | --- | --- |
| Infant Toddler (Checklist (ITC) [1] | Parent/carer | 6-24 months | 24 | Less than 5 minutes |
| Quantitative Checklist for Autism in Toddlers (Q-CHAT) (2r) [2] | Parent/carer | 18-24 months | 25 likert-type items | Less than 10 minutes |
| Modified Checklist for Autism in Toddlers- Revised / with Follow-up (MCHAT R/F) [3] | Parent/carer | 16-30 months | 20 | Less than 10 minutes.-There is a follow up in-person or via phone structured interview for suspected cases |
| Checklist for Autism in Toddlers (CHAT) [4] | Parent/carer & General Practitioner or Health Care worker | 18 months | 9 items reported by parent/carer and 5 items based on professional observation | Less than 10 minutes |
| Early Screening for Autism Traits (ESAT) [5] | Parent/carer & Child Care worker | 14-15 months | 14 | 10 minutes |
| Checklist for Early Developmental Disorders (CESDD) [6] | Day-care staff | 3-36 months | 25 (Autism) + 4 (Language) | 10 minutes-Parents of children identified as at-risk must complete additional screeners |
| Social Attention Communication Behaviours Surveillance [7] | Nurse observation schedule | 20-48 months | 5 | Less than 10 minutes |
| Screening Tool for Autism in Toddlers and Young Children (STAT) [8] | Community service providers working with young children | 24-36 months | 12 interactive activities | 20 minutes |
| Pervasive Developmental Disorder Screening Test-II (PDDST-II) [9] | Parent/carerDifferent versions for primary care (I), developmental clinic (II), autism clinic (III) | 12-48 months | Version I: 22 itemsVersion II: 14 itemsVersion III: 12 items | Variable. Parents can complete a questionnaire or be interviewed |
| Communication and Symbolic Behavior Scales (CSBS) [10] | Parent/carer | 6-24 months | 24 | Less than 10 minutes |

References Table S1 (Screening Questionnaires for Young Children)

1. Wetherby A, Prizant B (2002). The Infant Toddler Checklist from the Communication and Symbolic Behavior Scales. Baltimore: Brookes Publishing.
2. Allison C, Baron-Cohen S, Wheelwright S, Charman T, Richler J, Pasco G, Brayne C. (2008). The Q-CHAT (Quantitative Checklist for Autism in Toddlers): A Normally Distributed Quantitative Measure of Autistic Traits at 18–24 Months of Age: Preliminary Report. Journal of Autism and Developmental Disorders 38: 1414–1425.  doi: 10.1007/s10803-007-0509-7
3. Robins et al. (2009; corrected version 2018). Checklist for Autism in Toddlers, Revised, with Follow-Up. (M-CHAT-R/F), https://mchatscreen.com
4. Baron-Cohen S et al. (2000). The early identification of autism: The Checklist for Autism in Toddlers (CHAT). Journal of the Royal Society of Medicine 93: 521-525. doi: [10.1177/014107680009301007](https://doi.org/10.1177/014107680009301007)
5. Swinkles SH, Dietz C, van Daalen E, Kerkoff IH, van Engeland H, Buitelaar JK. (2006). Screening for autistic spectrum in children aged 14 to 15 months. I: the development of the Early Screening of Autistic Traits Questionnaire (ESAT). J Autism Dev Disord 6:723-32. doi: 1[0.1007/s10803-006-0115-0](https://doi.org/10.1007/s10803-006-0115-0)

# Dereu M, Warreys P, Raymaekers R, Meirsschaut M, Pattyn G, Schietecatte I, Roeyers H. (2010). Screening for autism spectrum disorders in Flemish day-care centres with the checklist for early signs of developmental disorders. J Autism Dev Disord 40:1247-58. doi: 10.1007/s10803-010-0984-0

# Barbaro J, Ridgway L, Dissanayake C. (2011). Developmental surveillance of infants and toddlers by maternal and child health nurses in an australian community-based setting: promoting the early identification of autism spectrum disorders. Journal of Pediatric Nursing 26, 334–347. doi:10.1016/j.pedn.2010.04.007

1. Stone W et al. The STAT - Screening Tool for Autism in Toddlers & Young Children. Vanderbilt University [Center for Technology Transfer and Commercialization](http://g.co/maps/x4k3z). http://stat.vueinnovations.com/about
2. Montgomery J, Duncan, C, C. Francis, G. (2007). Test review: Siegel B. (2004). Pervasive developmental disorder screening test-II (PDDST-II). San Antonio, TX: Harcourt. J Psychoeduc Assess 25: 299-306. 10. doi:1177/0734282906298469
3. Prizant B, Wetherby A. (2001). Communication and Symbolic Behavior Scales Developmental Profile (CSBS), Baltimore, MD: Brookes Publishing.

# *Table S2 - Examples of frequently used screening questionnaires for older children and/or adults*

| *Scale* | *Informant* | *Age range* | *Number of items* | *Time to administer* |
| --- | --- | --- | --- | --- |
| Social Communication Questionnaire (SCQ) [1] | Parent /carer | 36-82 months | 40 | 15-20 minutes |
| Childhood Autism Spectrum Test (CAST) [2] | Parent/carer | 5-11 years | 39 | 10 minutes |
| Social Responsiveness Scale (SRS) [3] | Parent/carer | 4-18 years | 65 | 15 minutes |
| Autism Spectrum Screening Questionnaire (ASSQ) (3r) [4] | Parent-carer/teacher) | 6-17 | 27 | Less than 10 minutes |
| Children's Social Behavior Questionnaire (CSBQ) (4r) [5] | Parent/carer | 4-18 | 49 | Less than 15 minutes |
| Social Communication Disorders Checklist (SCDC) (5r) [6] | Parent-carer/ teacher | 3-19 | 12 | Less than 5 minutes |
| The Autism - Tics, AD/HD and other Comorbidities inventory (A-TAC) (6r) [7] | Interviewer based screening telephone with parents. | 7-18 | 178 item interview | Average 27,5 minutes |
| Autism Spectrum Quotient (AQ) [8] | Self-report by a person without intellectual disability | Adulthood | 50-A shorter 10 items adaptation is available as an informant version for adolescents | Variable |
| Ritvo Autism Asperger Diagnostic Scale-Revised (RAADS-R) [9] | Administered by a clinician in a clinical setting | Adulthood | 80 | Variable |

*References Table S2* (Screening questionnaires for older children and adults)

1. Rutter M, Bailey A, Lord C. (2003). Social Communication Questionnaire (SCQ). California: Western Psychological Services.Constantino J. (2005). Social Responsiveness Scale (SRS). California: Western Psychological Services.
2. Scott F, Baron-Cohen S, Bolton P, Brayne C. (2002). The CAST (Childhood Asperger Syndrome Test): Preliminary development of UK screen for mainstream primary school children. Autism 6:9-31. doi:[10.1177/1362361302006001003](https://doi.org/10.1177/1362361302006001003)
3. Constantino J. (2005). Social Responsiveness Scale (SRS). California: Western Psychological Services.
4. Baron-Cohen S et al. (2001). The Autism Spectrum Quotient (AQ) The Autism Research Center. https://www.autismresearchcentre.com/arc_tests
5. Hartman C., de Bildt A., Minderaa R. (2013) CSBQ (Children’s Social Behavior Questionnaire). In: Volkmar F.R. (eds) Encyclopedia of Autism Spectrum Disorders. Springer, New York, NY
6. Skuse DH, Mandy WP, Scourfield J. (2005). Measuring autistic traits: heritability, reliability and validity of the Social and Communication Disorders Checklist. The British Journal of Psychiatry187: 568-572.
7. Mårland, C., Lichtenstein, P., Degl’Innocenti, A. *et al.* (2017). The Autism–Tics, ADHD and other Comorbidities inventory (A-TAC): previous and predictive validity. *BMC Psychiatry* **17,**403. https://doi.org/10.1186/s12888-017-1563-0
8. 8. Baron-Cohen S et al. (2001). The Autism Spectrum Quotient (AQ) The Autism Research Center.
9. <https://www.autismresearchcentre.com/arc_tests. Accessed 5 May 2020>

9. Ritvo RA et al. (2011). The Ritvo Autism Asperger Diagnostic Scale - Revised (RAADS-R): A scale to assist the diagnosis of ASD in adults: an international validation study. J Autism Dev Disord 41:1076-1089. doi: [10.1007/s10803-010-1133-5](https://dx.doi.org/10.1007%2Fs10803-010-1133-5)

# *Table S3 – Examples of frequently used diagnostic interviews for autism*

| *Scale* | *Informant/* *Type of measure* | *Age range* | *Number of items* | *Time required / Administrator* |
| --- | --- | --- | --- | --- |
| Autism Diagnostic Interview-Revised (ADI-R) [1] | Parent/carer-Semi-structured interview | Children and adults; mental age above 24 months | 93 | 90-150 minutes, including scoring-Interviewer: experienced clinician with specific training |
| Autism Diagnostic Observation  Schedule-(ADOS-2) [2] | -Semi-structured observational assessment | From 12 months, through adulthood | One of 5 modules needs to be chosen and applied (around 14 activities in each) | 40-60 minutes-Examiner: experienced clinician with specific training |
| The Diagnostic Interview for Social and Communication Disorders (DISCO) [3] | Parent/carer-Semi-structured interview; provides dimensional approach for assessment of symptoms and needs | Early years to adulthood | Over 300 items organized in 8 parts | 120-180 minutes-Interviewer: experienced clinician with specific training |
| Developmental Dimensional and Diagnostic Interview (3di) [4] | Parent/care-giver interview-Structured/semi structured computerised interview-Provides a structured report | Early years to adulthood | Full instrument contains 800 questions | 90 - 120 minutes-Short version 45 minutes-Minimal professional qualification, but specific training is required |
| Developmental, Dimensional and Diagnostic Interview-Adult Version (3Di-Adult).(7r) [5] | Informant (who has known adult since childhood) interview.Structured/semi structured computerised interview-Provides a structured report | Early years to adulthood | 69 questions, 48 current behaviour and 21 past behaviour. | Average 50 minutes.Minimal professional qualification, but specific training is required |
| Childhood Autism Rating Scale (CARS-2) [6] | Behavioural rating scale, completed after collecting information from a variety of people and situations, and from clinical observations - Information form for parent/carers also available | Early years to adulthood | 15 itemsA High Functioning (CARS-HF) version is also available | 10-15 minutes after data is collected-Requires an experienced clinician |

References Table S3 (Examples of diagnostic interviews for autism)

1. Lord C, [Rutter M](https://en.wikipedia.org/wiki/Professor_Sir_Michael_Rutter), Le Couteur A. (1994). Autism Diagnostic Interview-Revised: a revised version of a diagnostic interview for caregivers of individuals with possible pervasive developmental disorders. J Autism Dev Disord 24 (5): 659-85. [doi](https://en.wikipedia.org/wiki/Digital_object_identifier):[10.1007/BF02172145](https://doi.org/10.1007%2FBF02172145)
2. Lord C, Rutter M, DiLavore PC, Risi S, Gotham K, Bishop S, Luyster RJ, Guthrie W (ADOS-2) Autism Diagnostic Observation Schedule, Second Edition (2012). London: Pearson´s International
3. Wing L. Diagnostic Interview for Social and Communication Disorders (11th edn.) (2003). London: National Autistic Society
4. Skuse D, Warrington R, Bishop D, Chowdhury U, Lau J, Mandy W, Place M. (2004). The Developmental, Dimensional and Diagnostic Interview (3di): A novel computerized assessment for Autism Spectrum Disorders. Journal of the American Academy of Child and Adolescent Psychiatry 43: 548-58. doi:[10.1097/00004583-200405000-00008](https://doi.org/10.1097/00004583-200405000-00008)
5. Mandy W, Clarke K, McKenner M, Strydom A, Crabtree J, Lai MC, Allison C, Baron-Cohen S, Skuse D. (2018). Assessing Autism in Adults: An Evaluation of the Developmental, Dimensional and Diagnostic Interview—Adult Version (3Di-Adult). Journal of Autism and Developmental Disorders. 48(2):549-560. doi: 10.1007/s10803-017-3321-z.
6. Schopler E, Van Bourgondien ME. (2010). Childhood Autism Rating Scale-2 (CARS-2). Torrance, CA: Western Psychological Services.

# *Table S4. Examples of developmental, cognitive and adaptive behaviour measures*

| *Scale* | *Administration/*  *type of measure* | *Age range* | *Domains covered.* | *Time required / Administrator* |
| --- | --- | --- | --- | --- |
| Battelle Developmental Inventory Screening Test, 2nd (BDI-2) [1] | Individual administration; focus on general development. | Birth to 7 years 11 months | 5 global domains: personal-social, adaptive, motor, communication, cognitive skills; divided in 13 subdomains | Complete battery: 60-90 minutes  -Screener test:10-30 minutes  -Examiner: teachers and test professionals |
| Bayley Scales of Infant and Toddler Development, III [2] | Individual administration; focus on general development. | 1 to 42 months | Full scale covers` 5 domains: cognitive, language, motor, socio-emotional, adaptive behaviour.  A screening version covers cognitive, language and motor skills | - Full scale 30 to 90 minutes (depending on age of the child)  -Screening version 15-25 minutes  -Examiner: qualified professionals in child development  (Independent study training program can be purchased) |
| Merrill-Palmer Revised Scales of Development (M-P-R) [3] | Individual administration, plus parent/  examiner rating  - Focus on general development. Also appropriate for  children with hearing impairments, autism, other developmental delays/ disabilities | 1 to 78 months | Cognitive and Motor – are evaluated by examiner  Self-help /Adaptive, Language and Communication, and Social / Emotional behaviour – are evaluated by both examiner and parent | 45 minutes  -Examiner must have completed graduate-level courses in tests and measurement |
| Leiter International Performance Scale, Third Edition (Leiter-3) [4] | Individual administration.  -Completely non-verbal: designed for individuals who are cognitively delayed; non-English speaking, hearing or speech impaired, or on the autism spectrum.  -No cultural bias | 3 years  to 75 years | Covers 2 main areas: Cognitive / Fluid intelligence and Attention/Memory organized in 10 sub-tests.  -There is a complementary social-emotional examiner rating scale | 20-45 minutes  -Examiner: requires at least a Master degree in related professions |
| Wechsler Preschool and Primary Scale of Intelligence \| Fourth Edition (WPPSI-IV) [5] | Individual administration.  For verbal children  No cultural bias | Two age bands:  - 2:6 years to 3:11 years  and  - 4:0 years to 7.7 years | It generates a Full Scale IQ. It provides three primary index scores: Verbal Comprehension Index, Visual Spatial Index for first age band, and adds Processing Speed Index for the older children | 30-45 minutes for the younger and 45-60 minutes for the older children  -For all Wechsler tests, examiner should have appropriate training in test administration |
| Wechsler Intelligence Scale for Children \| Fifth Edition (WISC-V)[6] | Individual administration.  For verbal children.  No cultural bias | 6 years to 16 years 11 months | It generates a Full Scale IQ (formerly known as an intelligence quotient or [IQ score](https://en.wikipedia.org/wiki/Intelligence_quotient)) that represents a child's general intellectual ability. It also provides five primary index scores: Verbal Comprehension Index, Visual Spatial Index, Fluid Reasoning Index, Working Memory Index, and Processing Speed Index. | 45-65 minutes |
| The Wechsler Adult Intelligence Scale—Fourth Edition (WAIS-IV)[7] | Individual administration.  For verbal adults.  No cultural bias | 16-90 years | It generates a Full Scale IQ . There are four index scores representing major components of intelligence:VerbalComprehension Index (VCI), Perceptual Reasoning Index (PRI),Working Memory Index (WMI),Processing Speed Index (PSI) | 60-90 minutes |
| The Mullen Scales of Early Learning (8) [8] | Individual administration. | Birth to 68 months | 124 items 5 subscales: (a) gross motor, (b) fine motor, (c) visual reception (or non-verbal problem solving), (d) receptive language, and (e) expressive language. An early learning composite score can be derived from fine motor, visual reception, receptive language, and expressive language scales | 45 minutes |
| Vineland Adaptive Behavior Scales, Third Edition (Vineland-3) [8] | Interview and parent/carer form.  -Teacher form for 3 to 21 years old  -For individuals with intellectual and developmental disabilities, delays, autism and other impairments | Birth to 90 years | Three mandatory domains: Communication (receptive, expressive and written); Daily Living Skills (personal, domestic and community); Socialization (interpersonal relationships, play and leisure, coping skills)  -Two optional domains: motor and maladaptive behavior | 30 minutes (20 minutes for interview form and 10 minutes for parent/carer/teacher form  -Digital or paper-based administration |
| Adaptive Behavior Assessment System, Third Edition (ABAS-3) [9] | Parent/carer form (Ages 0-5); teacher/care provider form (Ages 2 to5); parent form (Ages 5 to 21); and teacher form (5 to 21)  -Adult form: can be completed by the individual  Proposes involving different people | Birth to 89 years  - Different versions for ages 0 to 5; 5 to 21, and 16 to 89 years | Covers conceptual, social and practical domains, by assessing 11 adaptive skill areas | 15-20 minutes  -Available in three formats: online, software and print  -Requires a Master degree in related professions |

# References Table S4 (Examples of developmental, cognitive and adaptive behaviour measures)

1. Newborg J. (2005). Battelle Developmental Inventory, 2nd Edition: Examiner’s manual. Itasca, IL: Riverside

2. Bayley N. (2005). Bayley Scales of Infant and Toddler Development | Third Edition. London: Pearson´s International

3. Roid G, Sampers J. (2004). Merrill-Palmer-Revised Scales of Development, Wood Dale, Illinois: Stoelting Co.

4. Roid G, Miller L, Pomplun M, Koch C. (2013). Leiter International Performance Scale third edition (Leiter-3), Wood Dale, Illinois: Stoelting Co.

5. Wechsler, D (2012). Wechsler Preschool and Primary Scale of Intelligence (4^th^ ed.). Bloomington: Pearson

6. Wechsler, D. (2014). ​WISC​-V: Technical and Interpretive Manual​. Bloomington, MN: Pearson.

7. Wechsler, D. (2008). WAIS-IV Administration and Scoring Manual. San Antonio, TX: The

Psychological Corporation.

8. Mullen, E. M. (1995). Mullen Scales of Early Learning (AGS ed.). Circle Pines, MN: American Guidance Service Inc.

9. Sparrow S, Cicchetti D, Saulnier C. (2016). Vineland Adaptive Behavior Scales Third Edition (Vineland-3). Minneapolis, MN: Pearson Assessment.

10. Harrison P, Oakland T. (2015). Adaptive Behavior Assessment System (ABAS-3) - Third Edition (ABAS-3). California: Western Psychological Services.

*Table S5 - Genetic etiological investigations in people with ASD*

| Steps |  |
| --- | --- |
| #1 | Referral for genetic evaluation. |
| #2 | Three-generation family history, with a particular focus on autism, other neurodevelopmental conditions, psychiatric or neurological traits |
| #3 | Physical examination: dysmorphic features, abnormal growth parameters (including head circumference), skin examination and neurological abnormalities. If a syndrome or metabolic disorder is suspected referred to appropriate specialist and/ or for specific genetic testing. |
| #4 | Laboratory testing, discuss and offer:  - CMA ( chromosomal microarrays) analysis. If not available, karyotyping.  - Fragile X analysis for all males and in females if suggestive of history of sex-linked  intellectual disability inheritance, especially if large head size, prominent jaw, large ears, ligamentous laxity, and, in males large testes after puberty.  - MECP2 gene testing for Rett syndrome if girl with significant loss of previous acquired skills, deceleration in head growth and/or midline hand movements.  - PTEN gene analysis if marked macrocephaly and in boys with pigmented macules on the penis.  If these studies do not reveal aetiology and there are dysmorphic features, severe or moderate intellectual disability and/or severe language delay proceed to step 5. |
| #5 | Advanced genetic testing: discuss and offer Whole Exome Sequencing (WES) [soon to be replaced by Whole Genome Sequencing (WGS)] |

- Adapted from Griesi-Oliveira K, Laurato Sertié A (2017) Autism spectrum disorders:

an updated guide for genetic counseling. Einstein (Sao Paulo) 15: 233-238 DOI: [10.1590/S1679-45082017RB4020](https://dx.doi.org/10.1590%2FS1679-45082017RB4020). Accessed 5 May 2020

*Table S6 – Examples of current interventions for autism*

| *Interventions with a focus on young children* | Examples *(Note: for each of these interventions there is some supportive evidence, although the evidence base for many is limited)* |
| --- | --- |
| Early parent mediated interventions to facilitate social communication skills.  Focus is on teaching parents/careers to establish joint engagement with the child. Over-directiveness by adult is discouraged with emphasis on: synchrony between adult and child; creating opportunities for shared attention; functional reciprocal communication (verbal or non-verbal); spontaneous play | Developmental Individual-Difference Relationship-Based Model (DIR) or Floortime [1]  Early Social Interaction (ESI) [2]    Early Start Denver Model (ESDM)* [3]  Joint Attention Symbolic Play Engagement and Regulation (JASPER)* [4]  Preschool Autism Communication Therapy (PACT) [5]  Hanen More than Words [6]  (* also, included in NDBI, see below) |
| Applied Behaviour Analysis (ABA)  A *general* approach to reduce problem behaviours, or enhance learning. Involves functional analysis of potential causes of child’s difficulties and use of behavioural principles to increase skills (reinforcement, prompting, modelling and shaping etc.) | *For examples of specific programmes see EIBI and NDBI approaches (below)* |
| Early Intensive, behavioural interventions  Highly prescriptive programmes based on ABA principles involving up to 40 hours per week therapy based on ABA principles | Lovaas model of early intensive (home based) behavioural intervention (e.g. (EIBI) [7,8] |
| Early naturalistic developmental behaviour interventions (NDBI) Emphasis is on child play, social interaction and initiating communication. Based on behavioural strategies but following a more individually based, developmental perspective than EIBI programmes | Enhanced Milieu Teaching (EMT) [9]  Incidental Teaching (IT) [10]  Pivotal Response Treatment (PRT) [11]  Reciprocal Imitation Training (RIT) [12]  Social Communications/Emotional Regulation/Transactional Support (SCERTS) [13] |
| Parent focused management programmes | Pre-schoolers with Autism: Parent Education and Skills Training Programme. [14]  Stepping Stones Triple P [15]  German Manualized Treatment Approach [16] |
| *Approaches with a focus on older children* |  |
| Interventions with a focus on structure; predictability and/or verbal/non-verbal cues | TEACCH [17]  Social Stories [18]  Picture Exchange Communication System (PECS) [19] |
| Social skills interventions to improve peer relationships & communication (mainly school based) | Secret Agent Society [20]  PEERS program [21] |
| *Approaches with a focus on adolescents and adults* |  |
| Interventions to improve mental health | Cognitive behaviour therapy [22,23,24]  Mindfulness [25] |
| Interventions to improve social integration | Social skills learning [20, 21,26,27,28]    Supported employment [29,30,31,32]  Recreational, daily living skills and leisure programmes [33] |

References Table S6 (Examples of current interventions for autism)

1. The Interdisciplinary Council on Development and Learning, Inc. DIR, Floortime, and the DIRFloortime. <http://www.icdl.com/home>. Accessed 5 May 2020

2. Wetherby AM, Guthrie W, Woods J, Schatschneider C, Holland RD, Morgan L, Lord C. (2014). Parent-implemented social intervention for toddlers with autism: an RCT. Pediatrics 134:1084-1093. doi:10.1542/peds.2014-0757

3. Rogers S, (2016). Early Start Denver Model. In *Comprehensive models of autism spectrum disorder treatment* (pp. 45-62). Springer, Cham.

4. Kasari C. (JASPER). Joint Attention, Symbolic Play, Engagement and Regulation. <https://www.jetsstudy.org/jasper>. Accessed 5 May 2020

5. Pickles A, Le Couteur A, Leadbitter K, Salomone E, Cole-Fletcher R, Tobin H, Gammer I, Lowry J, Vamvakas G, Byford S, Aldred C, Slonims V, McConachie H, Howlin P, Parr J, Charman Y, Green K. (2016). Parent-mediated social communication therapy for young children with autism (PACT): long-term follow-up of a randomised controlled trial. The Lancet 388: 2501-2509. <https://doi.org/10.1016/S0140-6736(16)31229-6>

6. More Than Words — The Hanen Program for Parents of Children with Autism Spectrum Disorder or Social Communication Difficulties. <http://www.hanen.org/Programs/For-Parents/More-Than-Words.aspx>. Accessed 5 May 2020

# 7. Lovaas OI. (1987). Behavioral treatment and normal educational and intellectual functioning in young autistic children. J Consult Clin Psychol 55: 3-9

8. Caron V, Bérubé A, Paquet A. (2017). Implementation evaluation of early intensive behavioral intervention programs for children with autism spectrum disorders: A systematic review of studies in the last decade. Eval Program Plann 62: 1-8. doi: 10.1016/j.evalprogplan.2017.01.004

9. Hancock TB, Kaiser AP. (2002). The effects of trainer-implemented enhanced milieu teaching on the social communication of children with autism. Topics in Early Childhood Special Education, 22: 39–54. <https://doi.org/10.1177/027112140202200104>

10. McGee GG, Morrier MJ, Daly T. (1999). An incidental teaching approach to early intervention for toddlers with autism. Journal of the Association for Persons with Severe Handicaps, 24: 133–146. <https://doi.org/10.2511/rpsd.24.3.133>

11. Verschuur R, Didden R, Lang R, Sigaffos J, Huskens B. (2014). Pivotal response treatment for children with autism spectrum disorders: a systematic review, J Autism Dev Disord 1:34–61. doi: 10.1007/s40489-013-0008-z

12. Ingersoll, Brooke & Schreibman, Laura. (2006). Teaching Reciprocal Imitation Skills to Young Children with Autism Using a Naturalistic Behavioral Approach: Effects on Language, Pretend Play, and Joint Attention. Journal of autism and developmental disorders. 36. 487-505. doi:10.1007/s10803-006-0089-y.

13. Prizant B, Wetherby A, Rubin E, Laurent A, Rydell P. (2006). The SCERTS Model: A Comprehensive Educational Approach for Children with Autism Spectrum Disorders. Baltimore, MD: Paul H. Brookes Publishing.

14. Tonge B, Brereton A, Kiomall M, Mackinnon A, Rinehart NJ. (2014). A randomised group comparison controlled trial of ‘preschoolers with autism’: A parent education and skills training intervention for young children with autistic disorder. Autism 18: 166–177. <https://doi.org/10.1177/1362361312458186>

15. Hodgetts S, Savage A, McConnell D. (2013). Experiences and outcomes of stepping stones triple P for families of children with autism, Res Dev Disabil 34: 2572-85. doi: 10.1016/j.ridd.2013.05.005

16. Freitag CM, Jensen K, Teufel K, et al. (2020) Empirisch untersuchte entwicklungsorientierte und verhaltenstherapeutisch basierte Therapieprogramme zur Verbesserung der Kernsymptome und der Sprachentwicklung bei Klein- und Vorschulkindern mit Autismus-Spektrum-Störungen [Empirically based developmental and behavioral intervention programs targeting the core symptoms and language development in toddlers and preschool children with autism spectrum disorder] [Article in German] Z Kinder Jugendpsychiatr Psychother 48:224‐243. doi:10.1024/1422-4917/a000714

17. The University of North Carolina TEACCH Autism Program. Accessed 25 June 2019. <https://teacch.com>. Accessed 5 May 2020

18. Gray C. (2015). The New Social Story Book. Arlington, TX: Future Horizons

19. Flippin M, Reszka S, Watson LR. (2010). Effectiveness of the Picture Exchange Communication System (PECS) on communication and speech for children with autism spectrum disorders: a meta-analysis. Am J Speech Lang Pathol 19:178-95. doi: 10.1044/1058-0360(2010/09-0022)

20. Secret Agent Society. <https://www.sst-institute.net>. Accessed 5 May 2020

21. Laugeson EA, Frankel F, Gantman A, Dillon AR, Mogil C. (2012). Evidence-based social skills training for adolescents with autism spectrum disorders: the UCLA PEERS program. J Autism Dev Disord 42: 1025-36. doi: 10.1007/s10803-011-1339-1

22. Lorenc T, Rodgers M, Marshall D, Melton H, Rees R, Wright K, Sowden A. (2018). Support for adults with autism spectrum disorder without intellectual impairment: Systematic review. Autism, 22: 654-668. doi.org/10.1177/1362361317698939

23. White SW, Simmons GL, Gotham KO, Conner CM, Smith IC, Beck KB, Mazefsky C. (2018). Psychosocial treatments targeting anxiety and depression in adolescents and adults on the autism spectrum: review of the latest research and recommended future directions, Curr Psychiatry Rep 20: 82 https://doi.org/10.1007/s11920-018-0949-0

24. Spain D, Sin J, Harwood L, Mendez MA, Happé F. (2017). Cognitive behaviour therapy for social anxiety in autism spectrum disorder: a systematic review. Advances in Autism 3: 34-46. doi.org/10.1108/AIA0720160020

25. Sizoo B, Kuiper E. (2017). Cognitive behavioural therapy and mindfulness based stress reduction may be equally effective in reducing anxiety and depression in adults with autism spectrum disorders. Research in Developmental Disabilities 64:47-55. doi.org/10.1016/j.ridd.2017.03.004.

26. Einfeld SL, Beaumont R, Clark T, Clarke KS, Costley D, Gray K M, Siân KH, Redoblado MA, Roberts J, Sofronoff K, Taffe JR, Howlin, P. (2018). School-based social skills training for young people with autism spectrum disorders. Journal of Intellectual and Developmental Disability 43: 29-39. https://doi.org/10.3109/13668250.2017.1326587

27. Knonig C, Magill J, Volden J, Dick B. (2013). Efficacy of cognitive behavior therapy-based social skills intervention for school-aged boys with autism spectrum disorders. Research in Autism Spectrum Disorders 7: 1282–1290. doi:10.1016/j.rasd.2011.07.011

28. Gates JA, Kang E, Lerner MD. (2017). Efficacy of group social skills interventions for youth with autism spectrum disorder: A systematic review and meta-analysis, Clin Psychol Rev 52:164-181. doi: 10.1016/j.cpr.2017.01.006

29. Harmuth E, Silletta E, Bailey A, Adams T, Beck C, Barbic S. (2018). Barriers and facilitators to employment for adults with autism: a scoping review. Annals of International Occupational Therapy 1: 31-40. doi: 10.3928/24761222-20180212-01

30. Hedley D, Uljarević M, Cameron L, Halder S, Richdale A, Dissanayake C. (2017). Employment programmes and interventions targeting adults with autism spectrum disorder: A systematic review of the literature. Autism 21: 929– 941. doi.org/10.1177/1362361316661855

31. Mavranezouli I, Megnin-Viggars O, Cheema N, Howlin P, Baron-Cohen S, Pilling S. (2014). The cost-effectiveness of supported employment for adults with autism in the United Kingdom. Autism 18: 975–984. [doi.org/10.1177/1362361313505720](https://doi.org/10.1177/1362361313505720)

32. Wehman P, Schall C, McDonough J, Simma A, Brooke W, Ham H, Whittenbrurg V, Brooke L, Avellone E, Riehle E. (2019). Competitive employment for transition-aged youth with significant impact from autism: a multi-site randomized clinical trial. J Autism Dev Disord, p. 1-16. doi.org/10.1007/s10803-019-03940-2

33. Bishop‐Fitzpatrick L, Smith DaWalt L, Greenberg JS, Mailick MR. (2017). Participation in recreational activities buffers the impact of perceived stress on quality of life in adults with autism spectrum disorder. Autism Research 10: 973-982. doi:[10.1002/aur.1753](https://doi.org/10.1002/aur.1753)
